# Dissociation and its biological and clinical associations in functional neurological disorder: systematic review and meta-analysis

**DOI:** 10.1192/bjo.2022.597

**Published:** 2022-12-01

**Authors:** Malcolm C. Campbell, Abigail Smakowski, Maya Rojas-Aguiluz, Laura H. Goldstein, Etzel Cardeña, Timothy R. Nicholson, Antje A. T. S. Reinders, Susannah Pick

**Affiliations:** Institute of Psychiatry, Psychology & Neuroscience, King's College London, UK; and Central and North West London NHS Foundation Trust, London, UK; Institute of Psychiatry, Psychology & Neuroscience, King's College London, UK; and University Medical Center Hamburg-Eppendorf, Hamburg, Germany; Institute of Psychiatry, Psychology & Neuroscience, King's College London, UK; Center for Research on Consciousness and Anomalous Psychology (CERCAP), Department of Psychology, Lund University, Lund, Sweden

**Keywords:** Functional neurological disorder, conversion disorder, psychogenic non-epileptic seizures, dissociative disorders, dissociation

## Abstract

**Background:**

Studies have reported elevated rates of dissociative symptoms and comorbid dissociative disorders in functional neurological disorder (FND); however, a comprehensive review is lacking.

**Aims:**

To systematically review the severity of dissociative symptoms and prevalence of comorbid dissociative disorders in FND and summarise their biological and clinical associations.

**Method:**

We searched Embase, PsycInfo and MEDLINE up to June 2021, combining terms for FND and dissociation. Studies were eligible if reporting dissociative symptom scores or rates of comorbid dissociative disorder in FND samples. Risk of bias was appraised using modified Newcastle–Ottawa criteria. The findings were synthesised qualitatively and dissociative symptom scores were included in a meta-analysis (PROSPERO CRD42020173263).

**Results:**

Seventy-five studies were eligible (FND *n* = 3940; control *n* = 3073), most commonly prospective case–control studies (*k* = 54). Dissociative disorders were frequently comorbid in FND. Psychoform dissociation was elevated in FND compared with healthy (*g* = 0.90, 95% CI 0.66–1.14, *I*^2^ = 70%) and neurological controls (*g* = 0.56, 95% CI 0.19–0.92, *I*^2^ = 67%). Greater psychoform dissociation was observed in FND samples with seizure symptoms versus healthy controls (*g* = 0.94, 95% CI 0.65–1.22, *I*^2^ = 42%) and FND samples with motor symptoms (*g* = 0.40, 95% CI −0.18 to 1.00, *I*^2^ = 54%). Somatoform dissociation was elevated in FND versus healthy controls (*g* = 1.80, 95% CI 1.25–2.34, *I*^2^ = 75%). Dissociation in FND was associated with more severe functional symptoms, worse quality of life and brain alterations.

**Conclusions:**

Our findings highlight the potential clinical utility of assessing patients with FND for dissociative symptomatology. However, fewer studies investigated FND samples with motor symptoms and heterogeneity between studies and risk of bias were high. Rigorous investigation of the prevalence, features and mechanistic relevance of dissociation in FND is needed.

Functional neurological disorder (FND) is characterised by alterations in motor and/or sensory function that are not explained by or not compatible with identifiable neuropathology, according to DSM-5.^1^ FND symptoms vary; traditionally they have been characterised by motor symptoms (denoted here as FND-motor; e.g. muscle weakness, paralysis, disordered movements), seizures (FND-seizures) and sensory symptoms (FND-sensory; e.g. numbness). Increasingly, subjective cognitive difficulties, dizziness and auditory problems are being recognised and classified as FND.^2^

Dissociative symptoms are frequently reported in FND. A recent meta-analysis demonstrated this, but was limited by inclusion of one singular measure of dissociation – thus a broader inclusion of dissociation measures is needed.^3^ Dissociation as a neurocognitive process has been theorised to underlie FND, reflecting its categorisation in ICD-11, where the official term is dissociative neurological symptom disorder;^2,4,5^ here, it is classified as a dissociative disorder, alongside several other dissociative disorders, including dissociative identity disorder (DID) and depersonalisation/derealisation disorder (DPDR).^2^ Nevertheless, the conceptualisation of FND as a dissociative disorder, with dissociation as a core underlying mechanism, is not universally accepted. There is a paucity of high-quality empirical evidence supporting the conclusion that dissociation is a causal process in the generation of FND symptoms. Furthermore, there is no reference to dissociation as a mechanism in the DSM-5 classification of FND and so there is currently a notable and unhelpful discrepancy between the two major classification systems. The critical need to resolve this discrepancy necessitates more rigorous examination of the prevalence and potential mechanistic role of dissociation in FND in future studies.

Dissociation can broadly be viewed as the loss of control or awareness of cognitive or physical processes that are normally readily controlled or engaged in conscious awareness. As a symptom it can manifest as memory disturbance, subjective disconnectedness from the self (depersonalisation) or external stimuli (derealisation), loss of bodily sensation and voluntary control, or altered sense of identity.^6–9^ Although dissociation is considered a natural response to certain triggers, such as sleep deprivation or extreme fear (especially threat to life), we focus here specifically on pathological dissociation.^8,10,11^ Dissociative symptoms are seen in a variety of psychiatric disorders;^3,4,12–15^ symptoms can be chronic and severely disabling.^3,8^

Dissociation has previously been partitioned into compartmentalisation and experiential detachment;^4,16^ however, recent evidence from network analyses of dissociative experiences has extended this to include absorption (becoming absorbed in an external stimulus or one's own imagination to the point of reduced awareness of self and surroundings) and depersonalisation/derealisation.^17,18^ In compartmentalisation, individuals lose the ability to govern processes or actions over which they would normally have control; it is theorised to underpin FND-seizures and dissociative amnesia, for example.^19^ In detachment, individuals ‘detach’ from the ordinary sense of integration of self, body or external environment.^19^

Dissociative symptoms can also be categorised as somatoform symptoms, which are sensory (e.g. pain, loss or alteration in sensory modalities) or motor (e.g. weakness, involuntary movements), or psychoform, which relate to mental experiences such as memory impairment or depersonalisation.^20,21^ Various symptom rating scales screen for these clusters of symptoms. Table 1 describes some of the most commonly administered scales. The Somatoform Dissociation Questionnaire (SDQ-20), for example, primarily assesses somatoform dissociative symptoms,^20^ whereas the Dissociative Experiences Scale (DES) predominantly assesses psychoform dissociation.^22^ Little is currently known about the biological processes underpinning dissociation, and although several areas for further exploration have been highlighted recently,^15^ this is a crucial direction for future research to improve recognition, understanding and treatment of dissociative disorders.

**Table 1:** Example dissociation symptom rating scales

Rating scale	Developed by	Scoring	Description
Somatoform Dissociation Questionnaire (SDQ-20)	Nijenhuis et al (1996)^20^	20 items; 5-point Likert-like scale (1–5)	Measures trait somatoform dissociative symptoms Scores ≥35 are suggestive of a dissociative disorder Scores ≥50 are consistent with DID
Dissociative Experiences Scale (DES)	Bernstein & Putnam (1986)^22^	28 items; 0–100% for each	Measures trait psychoform dissociative symptoms Scores ≥30 are suggestive of a dissociative disorder
Multiscale Dissociation Inventory (MDI)	Briere (2002)^28^	30 items; 5-point Likert-scale (1–5)	Measures trait psychoform dissociative symptoms Has six subscales: Disengagement; Depersonalisation; Derealisation; Emotional constriction/numbing; Memory disturbance; Identity dissociation Patients scoring ≥15 on the Identity dissociation subscale likely to have DID
Clinician Administered Dissociative States Scale (CADSS)	Bremner et al (1998)^29^	27 items; 5-point Likert scale (0–4); clinician administered	Measures present-state dissociative symptoms Has three subscales: Depersonalisation; Derealisation; Amnesia
Dissociation Questionnaire (DIS-Q)	Vanderlinden et al (1993)^30^	63 self-report items; 5-point Likert scale (1–5)	Measures trait psychoform dissociative symptoms Has four subscales: Identity confusion; Loss of control over behaviour, thoughts and emotions; Amnesia; Absorption

DID, dissociative identity disorder.

Dissociative symptoms contribute to morbidity in psychiatric populations, especially in those who have experienced trauma. The dissociative subtype of post-traumatic stress disorder (PTSD), for example, has been associated with more severe illness.^23–25^ Individuals with a dissociative disorder are more likely to report self-harm and attempted suicide relative to other psychiatric populations, and psychiatric in-patients with a history of attempted suicide reported more severe dissociative symptoms than those without.^26^ Pathological dissociation contributes heavily to healthcare spending; however, timely diagnosis and treatment can mitigate this cost.^27^ Therefore, conditions involving significant dissociative symptom burden merit careful review with regard to risk assessment and management.

It is possible that dissociation is a negative mediator or prognostic marker in the overall clinical presentation and morbidity of FND. If true, a case can be made for broadening the treatment lens through which FND is managed by incorporating assessment and management of dissociative symptomatology. However, not enough is known about the extent of the role that dissociation plays in FND.

## Aims

The purpose of this systematic review and meta-analysis was to summarise the available evidence pertaining to dissociation in FND, as a symptom, comorbid disorder and potential prognostic marker. The primary aim was to critically appraise and report on rates of dissociative symptoms and disorders among people with FND. The secondary aim was to examine whether dissociation varies in severity in different FND symptom subgroups. The third was to report on the available data pertaining to biological and clinical associations of elevated dissociation in FND.

## Method

### Protocol registration

A protocol for this review was registered on PROSPERO on 24 April 2020 (ref CRD42020173263).

### Search strategy and study selection

The following databases were searched using the Ovid platform on 29 March 2020: Embase, PsycInfo and MEDLINE. The searches were updated on 1 June 2021. Studies were eligible for inclusion if they reported on comorbid dissociative disorder diagnoses and/or severity of dissociative symptoms in FND populations. The search terms for dissociation and FND are shown in the Appendix. Further articles were identified by reviewing the references of relevant systematic reviews, in addition to studies published during the selection process. The search strategy, including Boolean operators, is shown in supplementary Box 1, available at https://doi.org/10.1192/bjo.2022.597.

### Inclusion and exclusion criteria

The inclusion criteria were as follows:

- studies that reported on dissociative symptoms, as measured by validated rating scales (e.g. DES, SDQ-20)
- studies reporting on comorbid dissociative disorder diagnoses based on ICD or DSM criteria (e.g. using the Structured Clinical Interview for DSM Dissociative Disorders; SCID-D^31^)
- participants aged 18 years or older
- participants with specific FND diagnostic codes reflecting ICD or DSM criteria (supplementary Table 1)
- studies written in English, French, Spanish or Swedish
- studies published from 1 January 1980 or later, reflecting when DSM-III was released and the term ‘conversion disorder’ was adopted, replacing the outdated ‘hysteria’.

The exclusion criteria were:

- studies that explicitly stated that participants with FND had a comorbid major neurological diagnosis, e.g. FND-seizures with comorbid epilepsy
- studies in which participants with FND were included in mixed samples with additional psychiatric or physical health diagnoses, without presentation of disorder-specific dissociation data
- reviews and meta-analyses
- newspaper articles, editorials, non-peer reviewed sources, conference abstracts and other grey literature.

### Study screening

After removing duplicates, all titles and abstracts were screened by pairs of authors (M.C.C. and S.P. or A.S. and S.P.) and any that clearly did not meet the eligibility criteria were removed. The full texts of the remaining articles were then screened for eligibility by one author (M.C.C. or A.S.); reasons for study exclusion were documented.

### Data extraction

For each study, the following information was extracted and tabulated by one investigator (M.C.C. or A.S.), where available: FND sample (e.g. seizures, motor symptoms, mixed symptoms, unspecified), control group type (e.g. non-clinical, neurological, psychiatric), sample size, average age, gender ratio, dissociative symptom scales used and dissociative disorders diagnosed. Mean/median values for dissociation scales were recorded where available, in addition to measures of dispersion. Data from eligible studies that additionally used objective biological measures were also extracted, as were data on any clinical associations of dissociation in these studies.

### Quality appraisal

Studies were evaluated for quality and potential risk of bias by two independent raters (A.S. and M.R.-A.) using modified Newcastle–Ottawa criteria for case–control, cohort and cross-sectional studies.^32^ Discrepancies were discussed and resolved, with input from a third rater (S.P. or M.C.C.) where necessary. The criteria used are shown in supplementary Table 2.

### Synthesis method

The available data on rates of comorbid dissociative disorders, dissociative symptom scores and the clinical and biological associations of dissociation were first tabulated and synthesised qualitatively. We then conducted a meta-analysis of dissociative symptom scale scores. In the first meta-analysis, studies were included if they provided ‘adequate data’ (mean, standard deviation) for at least one validated measure of dissociative symptoms and control comparison. Studies with missing data were not included in the meta-analysis. A combined ‘psychoform dissociation measure’ was created by pooling data from the DES, Dissociation Questionnaire (DIS-Q) and Clinician Administered Dissociative States Scale (CADSS). The DES data were used for studies that reported more than one of these scales. The Somatoform Dissociation Questionnaire (SDQ) was calculated separately because it measures a different construct of somatoform dissociation.^20^ Studies that gave measures of central tendency as medians were excluded unless the authors also gave means and standard deviations. Studies that reported data from more than one dissociation measure or symptom subgroup were included in each measure group. Standard errors were converted to standard deviations according to Cochrane recommendations.^33^

Meta-analyses calculating dissociation scores used a random-effects model, with restricted maximum likelihood estimation^34^ to calculate heterogeneity variance τ^2^, Hartung–Knapp adjustments^35^ and Hedges’ *g* effect size metric, and were run on Windows 10 using the meta-R package^36^ with guidance.^37^ Funnel plots were generated to assess the risk of bias due to missing results. Meta regressions incorporating risk of bias categories were used to assess confidence in the body of evidence for each outcome.

We were able to explore the heterogeneity of our data by subgroup analyses because our meta-analyses contained more than ten studies. Subgroup analyses were conducted on studies that provided data for at least one FND group and a least one comparison group. The first subgroup analysis investigated dissociation scores by control group (healthy control, psychiatric and neurological). The second investigated the effect of FND symptom (seizures versus motor). Subgroup analyses applied a mixed-effects model using the subgroup function of the meta-R package.^36^ All meta-analyses are displayed in forest plots. Subgroup analyses were interpreted using Cochran's *Q*.^38^ *I^2^* heterogeneity statistics were interpreted using recommended levels.^39^

## Results

### Study selection

**Fig. 1:**
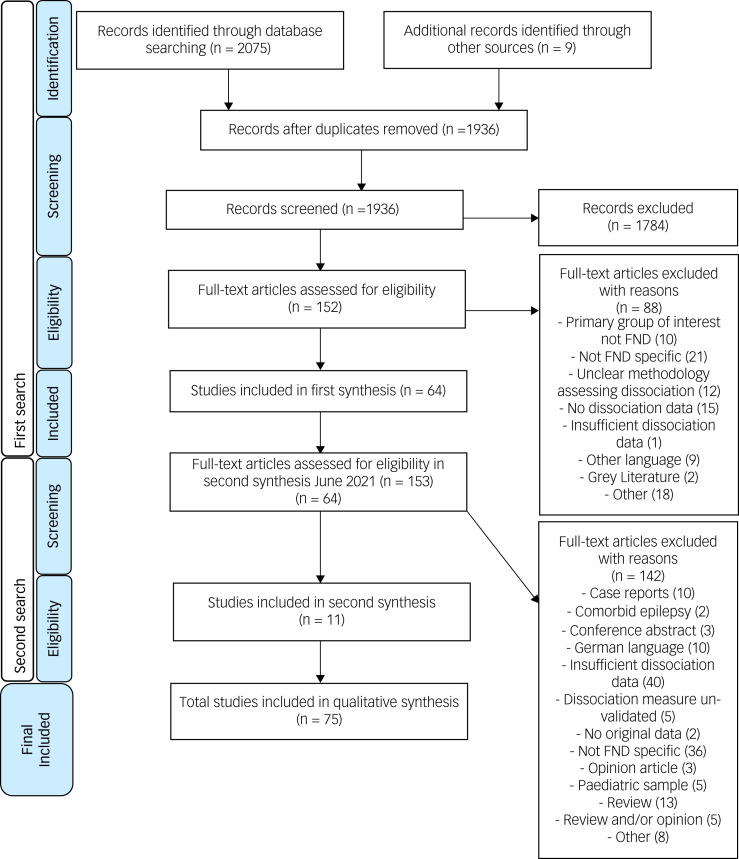
PRISMA flowchart. FND, functional neurological disorder.

The results of the study selection process are shown in the PRISMA flow diagram (Fig. 1). Seventy-five studies were included for qualitative review.

### Study characteristics

Details for each study – participant characteristics, design, principal findings, key strengths and weaknesses – are shown in supplementary Table 3. The majority of the included studies were case–control prospective studies (*k* = 54). FND samples were predominantly recruited in out-patient settings (*k* = 52). Of the FND participants, the most prevalent symptom profile reported was FND-seizures (*n* = 2160). FND-unspecified and FND-motor were the next most reported symptom types, with 956 and 523 participants respectively. Fifty-four studies included control groups. The most common control groups were healthy samples (*k* = 28), followed by epilepsy samples (*k* = 13). The mean age of FND participants ranged from 20.5 to 50.4 years. The female:male gender ratio ranged from 1.1:1 to 19.5:1; all studies that had male and female participants reported a greater female: male gender ratio. There were five studies with an all-female FND group and one with an all-male FND group.

### Quality appraisal and risk of bias

We used modified Newcastle–Ottawa Scale (NOS) criteria to rate the quality of all cohort and case–control studies.^40^ For cross-sectional studies, we applied an adapted version of the NOS.^32^ Overall, two of the case–control studies were rated as having a very high risk of bias, 32 a high risk of bias and 20 a low risk. Five cohort studies were rated as having high risk of bias; the sixth was at very high risk of bias. Ten cross-sectional studies were rated as high risk and the remaining five were at very high risk.

There were possible risks of bias identified across the studies; details of the key strengths and weaknesses of each study are shown in supplementary Tables 4–6. Examples of possible sources of bias observed in some studies include (but are not limited to) the following: small sample size; inadequate control for confounding variables (e.g. medication, age, severe psychiatric comorbidity); lack of control group(s); not explicitly stating that gold-standard diagnostic measures were used or not stipulating that diagnosis was made by a psychiatrist or neurologist; non-consecutive recruitment of FND participants.

### Comorbid dissociative disorders in FND samples

Table 2 presents the results of studies that reported on comorbid dissociative disorders in FND samples. The prevalence of comorbid dissociative disorders varied considerably across studies, with rates ranging from 8% to 80%. The highest proportion of individuals with a comorbid dissociative disorder was 80% in a sample of 10 participants with FND-seizures;^41^ the lowest was 8% in a group of 13 participants with FND-seizures.^42^ The most common comorbid dissociative disorder identified was dissociative disorder not otherwise specified (DDNOS); the least common was dissociative fugue. Diagnoses of dissociative amnesia, DPDR and DID were also reported in a proportion of participants in several studies.

**Table 2:** Subset of studies reporting on rates of comorbid dissociative disorders in samples with functional neurological disorder (FND)

Study	FND sample (*n*)	Participants with dissociative disorder, %	Dissociative disorder diagnosis	Diagnostic measure
Akyüz et al (2017)^43^	FND-unspecified (60)	48	Not stated	DSM-IV criteria
Baillés et al (2004)^44^	FND-seizures (30)	50	Dissociative amnesia (*n* = 3); DDNOS (*n* = 11); dissociative fugue (*n* = 1)	DSM-IV criteria
Litwin & Cardeña (2001)^41^	FND-seizures (10)	80	Dissociative amnesia (*n* = 4); DDNOS (*n* = 1); DPDR (*n* = 3)	DDIS
Marchetti et al (2009)^42^	FND-seizures (13)	8	DDNOS (*n* = 1)	DSM-IV criteria
Moene et al (2001)^45^	FND-unspecified (102)	10	DDNOS (*n* = 9); dissociative amnesia (*n* = 1)	DSM-III criteria
Roelofs et al (2002)^46^	Multiple (50)	26	Not stated	SCID-D
Scévola et al (2013)^47^	FND-seizures (35)	37	Not stated	DSM-IV criteria
Tezcan et al (2003)^48^	Multiple (59)	31	DDNOS (*n* = 8); DID (*n* = 9); dissociative amnesia (*n* = 1)	SCID-D DDIS
Yayla et al (2015)^14^	FND-unspecified (54)	37	DDNOS (*n* = 10); dissociative amnesia (*n* = 8); DPDR (*n* = 2)	SCID-D

DDIS, Dissociative Disorders Interview Schedule; DDNOS, dissociative disorder not otherwise specified; DID, dissociative identity disorder; DPDR, depersonalisation/derealisation disorder; SCID-D, Structured Clinical Interview for DSM Dissociative Disorders.

### Dissociative symptom scale scores in FND

Table 3 details the reported scores for the subset of studies that used validated dissociative symptom scales and presented measures of central tendency. A small subset of studies (*k* = 5) did not present measures of central tendency and are detailed only in supplementary Table 3.

**Table 3:** Dissociative symptom scale scores in samples with functional neurological disorder (FND)

Study	FND sample (*n*)	Control group (*n*)	Dissociation measure(s)	FND dissociation score, mean (s.d.)^a^	Control dissociation score, mean (s.d.)^a^
Akyüz et al (2004)^49^	FND-seizures (33)	Epilepsy (30)	DES	29.9 (20.1)	17.6 (15.6)
			CADSS	20.2 (9.9)	22.1 (3.7)
Akyüz et al (2017)^43^	FND-unspecified (60)		DES	23.9 (14.3)	
Alper et al (1997)^50^	FND-seizures (132)	Epilepsy (169)	DES	15.1 (13.5)	12.7 (10.8)
Bodde et al (2007)^51^	FND-seizures (22)		DIS-Q-1	1.2 (0.4)	
			DIS-Q-2	1.6 (0.6)	
			DIS-Q-3	1.7 (0.8)	
			DIS-Q-4	1.6 (0.6)	
Boesten et al (2019)^52^	FND-seizures; history of trauma (148)		TSI	62.9 (13.2)	
	FND-seizures; No history of trauma (69)		TSI	56.9 (13.6)	
Brown et al (2013)^53^	FND-seizures (43)	Epilepsy (24)	SDQ-20	**37.0 (17.0)**	**24.0 (7.0)**
Cope et al (2017)^54^^b^	FND-seizures without comorbid epilepsy (16)		DES^b^	18.7 (14.3)	
del Río-Casanova et al (2018)^55^	FND-unspecified (43)	Healthy control (42)	DES	**13.2**	**3.75**
			SDQ-20	**36**	**20**
Demartini et al (2016)^56^	FND-seizures (20)	Healthy control (20)	DES	17.2 (10.6)	8.2 (7.5)
			SDQ-20	23.3 (9.4)	22.8 (6.9)
			CDS	47.5 (46.8)	18.0 (28.7)
Demartini et al (2017)^57^	FND-motor (20)	Healthy control (20)	DES	9.9 (14.0)	3.2 (3.2)
		Anorexia nervosa (20)			11.5 (11.9)
Ekanayake et al (2017)^58^	FND-seizures (43)	Healthy control (26)	DES	15.9 (12.2)	n/a
		PMD (59)			5.6 (5.1
	FND-motor (59)		DES	5.6 (5.1)	
Espirito-Santo et al (2007)^59^	Multiple (25)	Dissociative disorder (36)	SDQ-20	39.8 (14.1)	39.3 (12.0)
		PTSD (49)			38.7 (11.7)
		MixP (116)			29.2 (6.7)
	FND-sensory (12)		SDQ-20	32.3 (5.2)	
	FND-motor (10)		SDQ-20	43.1 (16.8)	
	FND-mixed (3)		SDQ-20	58.7 (7.2)	
Espirito-Santo et al (2009)^60^	Multiple (26)	Dissociative disorder (39)	DES	43.5 (12.2)	36 (10.1)
		Somatisation disorder (40)			19.4 (11.9)
		MixP (46)			18.0 (8.4)
		Dissociative disorder (39)	SDQ-20	39.8 (14.2)	39.3 (11.9)
		Somatisation disorder (40)			31.8 (9.2)
		MixP (46)			29.6 (7.1 )
Evren & Can (2007)^61^	Multiple (55)		DES	28.8 (12.9)	
Gerhardt et al (2021)^62^	FND-seizures (44)	Healthy control (44)	SDQ-20	35.5 (7.8)	21.3 (2.0)
Goldstein et al (2000)^63^	FND-seizures (20)	Healthy control (20)	DES	22.6 (16.4)	13.1 (11.8)
			PAS	50.85 (7.24)	43.80 (14.73)
Goldstein et al (2006)^64^	FND-seizures (25)	Epilepsy (19)	DES	24.8 (16.5)	14.5 (10.2)
Gonzalez-Vazquez et al (2017)^65^	FND-unspecified (38)	MixP (292)	SDQ-20	37.2 (9.4)	27.0 (8.4)
		Dissociative disorder (30)			40.6 (14.6)
Guz et al (2003)^66^	FND-sensory (5)		DES	7 (2)	
	FND-mixed (43)		DES	26 (23)	
	FND-seizures (23)		DES	27 (27)	
	FND-motor (24)		DES	12 (16)	
Guz et al (2004)^67^	Multiple (87)	Somatisation disorder (71)	DES	21.6 (24.5)	16.9 (17.5)
Güleç et al (2014)^68^	Multiple; previous suicide attempt (33)	Healthy control (50)	DES	36 (21.9)	13.3 (20.0)
	Multiple; no previous suicide attempt (61)		DES	23.0 (18.1)	
Hammond-Tooke et al (2018)^69^	Multiple (29)	Healthy control (29)	DES	14.1 (6)	6.5 (2 6)
Herrero et al (2020)^70^	FND-seizures (34)	Healthy control (34)	DES	26.5 (13.9)	8.8 (6.8)
Holper et al (2021)^71^	FND-Seizures, comorbid epilepsy (25)	Epilepsy (234)	WDS	83.7 (44.0)	79.4 (30.5)
		Diagnosis unclear (60)			79.6 (32.4)
	FND-Seizures, no-comorbid epilepsy (62)		WDS	37.2 (48.0)	
	Other non-epileptic events (30)		WDS	75.2 (41.0)	
Irorutola et al (2020)^72^	FND-seizures (41)	Healthy control (41)	FDS	35.8 (7.8)	2.8 (3.8)
Jalilianhasanpour et al (2019)^73^	Multiple (34)		DES	19.5 (14.3)	
Jungilligens et al (2020)^74^	FND-seizures (20)	Healthy control (20)	DES	16.7 (18.4)	10.0 (9.4)
			SDQ-20	30.1 (9.7)	21.2 (1.4)
Kienle et al (2017)^75^	FND-unspecified meeting criteria for co-occurring PTSD (20)	PTSD (39)	DES	**19.6**	**38.6**
		Healthy control (40)			**6.5**
		PTSD (39)	SDQ-20	**36.5**	**36**
		Healthy control (40)			**21**
	FND-unspecified not meeting criteria for co-occurring PTSD (40)		DES	12.1	
			SDQ-20	28.5	
Koreki et al (2020)^76^	FND-seizures (41)	Healthy control (30)	SDQ-20	38 (12.8)	21 (0.0)
			MDI-DP.	11 (6.4)	5 (5.5)
Kranick et al (2011)^77^	FND-motor (53)	Movement disorder (22)	DES	6.4 (6.5)	5.6 (5.1)
		Healthy control (36)			4.9 (10)
Kuyk et al (2008)^78^	FND-seizures (22)		DIS-Q	1.9 (0.4)	
Litwin & Cardeña (2001)^41^	FND-seizures (10)	Epilepsy (31)	DES	**21.8**	**11.1**
Martino et al (2018)^79^	FND-seizures (10)	MDD (10)	DES	17.4 (17.4)	8.4 (13.8)
			SDQ-20	27.3 (10.3)	3.9 (3.5)
Martino et al (2021)^80^	FND-seizures; history of sexual abuse (15)		DES	34.6 (26.4)	
			SDQ-20	51.7 (14.9)	
	FND-Seizures; no history of sexual abuse (48)		DES	14.2 (12.5)	
			SDQ-20	42.3 (14.4)	
Mitchell et al (2012)^81^	FND-seizures (39)		DES	**20.7**	
Moene et al (2001)^45^	FND-unspecified (102)	Healthy control (89)	DIS-Q	1.7 (0.6)	1.5 (0.4)
		MixP (278)			2.1 (0.6)
Mousa et al (2021)^82^	FND-seizures (17)	Healthy control (20)	DES	86.6 (51.5)	23.9 (20.3)
Myers al (2019)^83^	FND-seizures (161)	Epilepsy (intractable) (96)	TSI	58.2 (12.7)	58.1 (14.4)
Nistico et al (2020)^84^	FND-seizures (11)	Healthy control (18)	CADSS-Total	19.7 (12.6)	7.8 (8.2)
			CADSS-DR	10.9 (6.3)	6.2 (5)
			CADSS-DP	5.9 (6.1)	1.9 (3.3)
			CADSS-DA	2.9 (2.0 )	0.9 (1.1)
	FND-motor (17)		CADSS-Total	15 (12.9)	
			CADSS-DR	9.4 (7.7)	
			CADSS-DP	6.9 (7.3)	
			CADSS-DA	2.1 (2.3)	
Ozcetin et al (2009)^85^	FND-seizures (56)	Healthy control (59)	DIS-Q	3.1 (0.8)	1.8 (0.5)
Ozdemir et al (2020)^86^	FND-unspecified (55)	Healthy control (45)	SDQ-20	43.4 (14.7)	25.7 (5.8)
O'Brien et al (2015)^87^	FND-seizures (19)	Healthy control (19)	DES	18.5 (16.7)	8.7 (6.2)
Perez et al (2018)^88^	Multiple (26)	Healthy control (27)	DES	19.7 (13.9)	3.3 (2.5)
			SDQ-20	33.5 (10)	20.0 (0.2)
Pick et al (2020)^89^	FND-mixed (19)	Healthy control (20)	CADSS-Total	**6 (8.5)**	**0 (1.0)**
			CADSS-DR^b^	**3 (5)**	**0 (0.75)**
			CADSS-DP^b^	**1 (3)**	**0 (0.75)**
			CADSS-DA^b^	**1 (4)**	**0 (0)**
Pick et al (2017)^90^	FND-seizures (37)	Healthy control (43)	SDQ-20	**34 (8)**	**21 (2)**
			MDI-Dis	**80 (24)**	**60 (16)**
			MDI-DP	**82 (62)**	**47 (9)**
			MDI-DR	**68 (44)**	**46 (11)**
			MDI-EN	**63 (38)**	**46 (4)**
			MDI-ME	**90 (57)**	**52 (19)**
			MDI-ID	**47 (47)**	**47 (0)**
Proença et al (2011)^91^	FND-seizures (20)	Epilepsy (20)	DES	54.3 (23.2)	22.0 ( 16.4)
Reedjik et al (2008)^92^	FND-unspecified (54)	Affective disorder (50)	DES	12 (10.9)	8.7 (7.1)
		CRPS (46)			6.6 (5.8)
		Affective disorder (50)	SDQ-20	30.7 (8.2)	23.6 (4.5)
		CRPS (46)			30.9 (9.7)
Reuber et al (2003)^93^	FND-seizures (98)	Epilepsy (63)	DES	17.2 (14.0)	8.8 (8.1)
Reuber et al (2003)^94^	FND-seizures – status epilepticus (33)	Epilepsy (64)	DES	18.6 (13.4)	8.8 (8.1)
Roelofs et al (2002)^95^	FND-unspecified (50)	MixP (50)	DES	11.7 (11,7)	9.1 (7.9)
			SDQ-20	30.5 (8.5)	23 (3.8)
			DIS-Q	1.8 (0.7)	1.8 (0.5)
Spinhoven et al (2004)^96^	FND-seizures (61)	CPP (52)	DES		8.6 (12.0)
			SDQ-20	28.8 (7.4)	25.7 (9.3)
			DIS-Q	0.5 (0.0)	
	FND-unspecified (102)		DIS-Q	1.7 (0.6)	
	FND-unspecified (54)		DES	12.0 (10.8)	
			SDQ-20	30.7 (8.2)	
			DIS-Q	1.9 (0.7)	
Spitzer et al (1999)^97^	FND-motor (16)		DES	15.9 (8.6)	
	FND-sensory (15)		DES	17.6 (9.8)	
	FND-seizures (21)		DES	15.3 (10.5)	
	Mix (20)		DES	17.5 (11.4)	
Steffen et al (2015)^98^	Multiple (45)	Healthy control (45)	SDQ-20	33.3 (9.5)	21.4 (1.7)
Steffen-Klatt et al (2019)^99^	Multiple (82)	Healthy control (82)	SDQ-20	**30 (9)**	**21 (2)**
Stins et al (2015)^100^	FND-unspecified (12)	Healthy control (12)	CADSS-DSS	8.4	**1.3**
Tezcan et al (2003)^48^	Multiple; all comorbid dissociative disorder (18)		DES	45.6 (14.1)	
	Multiple; no comorbid dissociative disorder (17)		DES	4.7 (2.5)	
	FND-mixed (7)		DES	20.6 (27.0)	
	FND-motor (5)		DES	25.0 (28.4)	
	FND-seizures (26)		DES	25.5 (16.4)	
	FND-sensory (21)		DES	17.4 (14.0)	
van der Hoeven et al (2015)^101^	FND-motor (55)	Healthy control (52)	SDQ-20	27.5 (7.2)	20.9 (1.5)
		Movement disorder (34)			24.4 (4.8)
		Healthy control (52)	DIS-Q	1.5 (0.3)	1.3 (0.2)
		Movement disorder (34)			1.3 (0.3)
van der Kruijs et al (2014)^102^	FND-seizures (21)	Healthy control (27)	SDQ-20	28.0 (6.8)	21.5 (4.7)
			DIS-Q	1.6 (0.4)	1.4 (0.2)
Walther et al (2019)^103^	FND-seizures; ongoing symptoms (33)		FDS	**21.5 (31.5)**	
	FND-seizures; symptoms ceased (19)		FDS	**6.5 (22)**	
Williams et al (2019)^104^	Multiple (56)		DES	19.2 (14.4 )	
			SDQ-20	32.2 (10.1)	
Wood et al (1998)^105^	FND-seizures (19)	Epilepsy (9)	DES	17.8 (11.2)	17.2 (16.4)
Xue et al (2013)^106^	FND-seizures (15)	Healthy control (15)	SDQ-20	**28 (4)**	**20 (0)**
Yayla et al (2015)^14^	FND-unspecified; dissociative disorder positive (20)		DES	29.3	
	FND-unspecified; dissociative disorder negative (34)		DES	9.1	

CADSS, Clinician-Administered Dissociative States Scale; CADSS-DA, CADSS Dissociative Amnesia; CADSS-DP, CADSS Depersonalisation; CADSS-DR, CADSS Derealisation; CPP, chronic pelvic pain; CADSS-DSS, CADSS Dissociative State Subscale; CDS, Cambridge Depersonalisation Scale; CRPS, Complex Regional Pain Syndrome; DES, Dissociative Experiences Scale; DIS-Q, Dissociation Questionnaire; DIS-Q-1, Identity confusion and depersonalisation; DIS-Q-2, DIS-Q Self-control; DIS-Q-3, DIS-Q Amnesia and dissociation; DIS-Q-4, DIS-Q absorption; FDS, Fragebogen zu Dissoziativen Symptomen; MDD, major depressive disorder; MDI, Multiscale Dissociation Index; MDI-DIS, MDI Disengagement; MDI-DP, MDI Depersonalisation; MDI-DR, MDI Derealisation; MDI-EN, MDI Emotional numbing; MDI-ID, MDI Identity dissociation; MDI-ME, MDI Memory disturbance; MixP, mixed psychiatric disorders; PAS, Perceptual Alteration Scale; PMD, psychogenic movement disorder; PTSD, post-traumatic stress disorder; SomD, somatisation disorder; SDQ-20, Somatoform Dissociation Questionnaire; TSI, Trauma Scale Index; WDS, Wessex Dissociation Scale;

a. Scores in bold show the median and interquartile range. All data are rounded to one decimal point where possible.

b. Data obtained directly from the author.

#### Dissociative Experiences Scale^22^

Thirty-nine studies presented DES scores; the mean DES score ranged from 4.71, in a sample of 17 individuals expressing multiple unspecified FND symptoms, to 86.6, in a sample of 17 with FND-seizures.^48,82^ FND-motor and FND-sensory samples endorsed lower DES scores in several studies.^48,57,58,66,77,97^ Participants without a comorbid dissociative disorder also reported lower DES scores.^14,48^ Conversely, participants with FND-seizures, mixed symptoms or comorbid dissociative disorders appeared to present with higher DES scores relative to the other FND subtypes.^14,48,49,64,66,80,82,91,96^ Although some of the highest scores on the DES were seen in FND-seizures samples (e.g.^82^), this was not a wholly consistent pattern as DES scores were low for this symptom type in some studies.^50,74,97^

Scores of 30 or more suggest severe pathological psychoform dissociation; this score is typically seen in dissociative disorders.^107^ Mean or median scores exceeded 30 in six separate samples: four with the FND-seizures subtype; two with a comorbid dissociative disorder; one with recorded prior suicide attempts; one with a history of sexual abuse; and one with multiple symptom types.^48,60,68,80,82,91^ A statistically significant elevation in DES scores was reported in FND samples relative to controls in 18 comparisons, with control groups including both clinical and non-clinical participants.^49,55–57,63,64,68–70,87,91,93,94^

#### Dissociation Questionnaire^30^

Eight studies measured dissociation using the Dissociation Questionnaire (DIS-Q).^45,51,78,85,95,96,101,102^ Five articles reported one or more control group scores, with seven control groups in total; the majority (four) were healthy control groups.^45,85,101,102^ DIS-Q scores were significantly greater in FND samples versus controls in four comparisons.^85,101,102^ However, DIS-Q scores varied without any clear trends across FND subtypes; in some cases, FND group DIS-Q scores were equal to or lower than control group scores.^45,95^

#### Trauma Symptom Inventory^108^

Two studies, both with participants with FND-seizures, used the Trauma Symptom Inventory (TSI); this includes a dissociation subscale.^52,83^ Boesten and colleagues^52^ observed significantly higher TSI dissociation scores in their cohort of patients with previous trauma compared with the non-traumatised cohort (*P* = 0.03), whereas Myers et al^83^ did not observe an appreciable difference in TSI dissociation scores when comparing people with epilepsy with people with FND-seizures (*P* = 0.97).

#### Cambridge Depersonalisation Scale^109^

Demartini and colleagues presented data for the Cambridge Depersonalisation Scale (CDS) in 20 individuals with FND-motor symptoms and 20 with FND-seizures.^56^ Depersonalisation as measured by this scale was significantly higher in the FND-seizures than the FND-motor group.

#### Clinician-Administered Dissociative States Scale^29^

Four studies presented state dissociation scores using the Clinician-Administered Dissociative States Scale (CADSS).^49,84,89,100^ Most scoring systems measure trait dissociation, whereas the CADSS measures state dissociation.^29^ Total CADSS scores were significantly higher in the FND group relative to controls in three studies.^84,89,100^ In one study the FND group CADSS score increased following a laboratory-based dissociation induction procedure.^89^ In one study, total CADSS score was higher in the epilepsy than in the FND-seizures group.^49^

#### Multiscale Dissociation Inventory^28^

Two studies used the Multiscale Dissociation Inventory (MDI).^76,90^ The MDI has six subscales measuring dissociation (depersonalisation, derealisation, amnesia, identity alterations, disengagement and emotional constriction).^28^ Pick et al^90^ included all subscales whereas Koreki and colleagues^76^ reported data for the depersonalisation subscale. Both studies investigated FND-seizures samples compared with healthy control groups; MDI scores for FND-seizures participants were significantly higher than for control groups in both studies. Pick et al reported statistically significant elevations in dissociation scores for all subscales. After controlling statistically for anxiety and depression, elevations remained significant for four subscales (disengagement, depersonalisation, derealisation and memory disturbance).^90^

#### Perceptual Alteration Scale^110^

Some studies did not report significantly elevated dissociation in FND samples. Goldstein et al measured dissociative tendencies using the Perceptual Alteration Scale (PAS) in 20 FND-seizures participants.^63^ Although the FND-seizures group had higher PAS scores than healthy controls, this difference was not statistically significant.

#### Wessex Dissociation Scale^111^

One study used the Wessex Dissociation Scale (WDS) in samples with pure FND-seizures, pure epilepsy, comorbid FND-seizures and epilepsy, ‘other non-epileptic events’ and unclear diagnoses.^71^ The pure FND-seizures group endorsed lower WDS scores than the other groups.

#### Fragebogen zu Dissoziativen Symptomen^112^

Two studies used the German-version of the DES, the Fragebogen zu Dissoziativen Symptomen (FDS).^72,103^ The FDS has included 16 new domains that are mainly FND symptoms and is thus not a pure psychoform measure. Walther and colleagues reported a significantly higher FDS score in FND-seizures participants with ongoing symptoms relative to those whose symptoms had abated.^103^ Irorutola et al observed a statistically significant elevation in FDS scores in FND-seizures participants relative to healthy controls.^72^

#### Somatoform Dissociation Questionnaire^20^

Twenty-four studies presented SDQ-20 scores (Table 3). A score of 35 or higher on the SDQ-20 is associated with dissociative disorders and suggests severe somatoform dissociation.^113^ Thirteen samples of FND participants had SDQ-20 scores ≥35.^53,55,59,60,65,75,76,80,86^ The highest scores were seen in an FND-motor sample, an FND-mixed sample, an FND-seizures sample (in which individuals who also reported previous sexual abuse scored far higher) and a sample of individuals with unspecified FND symptoms.^59,80,86^ Relative to other FND samples, several FND-seizures samples endorsed lower SDQ-20 scores; nevertheless, they still scored higher than the control groups in these studies.^56,79,102,106^ However, this was not consistent, as four FND-seizures groups had scores above 35.^53,62,76,80^

Twenty-two studies included control groups; some studies had multiple control groups, amounting to a total of thirty different control groups.^53,55,56,59,60,62,65,74–76,79,86,88,90,92,95,96,98,99,101,102,106^ Of these, five studies controlled for anxiety, depression or other psychopathology.^76,88,90,96,101^ In eighteen studies, SDQ-20 scores were statistically higher for FND participants relative to control groups.^53,55,59,60,65,74–76,79,86,88,90,95,98,99,101,102,106^ Conversely, SDQ-20 scores in FND groups were similar to or lower than scores in some control group comparisons, including PTSD, complex regional pain syndrome, dissociative disorders, chronic pelvic pain and one healthy control group.^56,59,60,75,92,96^

### Clinical characteristics relative to dissociation

Several studies examined the relationship between dissociation and severity of clinical outcome in FND-seizures samples. Statistically significant positive associations between dissociation scores and frequency of seizures were reported in five studies using various measures of dissociation.^51,78,80,87,103^ Two studies reported significant associations between dissociation scores and severity of ictal symptoms.^90,93^ Pick and colleagues observed that MDI depersonalisation and derealisation were positively associated with ictal mental state symptoms, whereas MDI identity dissociation was correlated with cognitive ictal symptoms; all of these associations were significant.^90^ Reuber and colleagues observed a weak but significant association between DES score and severity of ictal symptoms; this association was no longer significant after incorporating other psychopathological scores into the analysis.^93^

Two studies measured emotion dysregulation in FND participants using the Difficulties in Emotion Regulation Scale^114^ (DERS).^53,55^ Emotion dysregulation was positively correlated with psychoform and somatoform dissociation in an unspecified FND sample.^55^ More severe alexithymia and emotion dysregulation was reported in a subset of FND-seizures participants who scored higher on the SDQ-20 than the less emotionally dysregulated subgroup.^53^ Elsewhere, greater alexithymia severity was seen to vary with higher DES and SDQ-20 scores in a mixed FND sample.^75^ Quality of life of people with FND-seizures was significantly associated with dissociative symptoms in two studies.^81,115^ Both measured quality of life using the 31-item Quality of Life in Epilepsy Inventory (QOLIE-31).^116^ Individuals with higher degrees of dissociation measured by the DES had worse QOLIE-31 scores than those with lower DES scores. Two studies reported an association between history of sexual abuse, severity of clinical presentation and dissociation scores.^80,90^

One study found a significant association between psychological dissociation and psychopathology (general psychopathology, personality disorders) in an FND-motor group.^101^ DES scores were significantly higher in a sample with multiple FND symptoms who had attempted suicide than in a group who had not.^68^ Significant positive associations between severity of dissociation and various psychological features were reported in several samples of FND subtypes, including measures of alexithymia, emotion dysregulation, fearful attachment style and post-traumatic avoidance symptoms.^55,75,90,104,117^ Other associations included significant positive correlations between dissociation and number of FND symptoms, early onset of FND, in-patient treatment and rates of comorbid psychiatric illnesses.^14,45,96^

### Biological associations of dissociation in FND

The subset of studies that used objective means to study biological associations are described in Table 4.

**Table 4:** Studies reporting biological correlates of dissociation in functional neurological disorder (FND)

Study	FND sample (*n*)	Control group (*n*)	Investigation
Herrero et al (2020)^70^	FND-seizures (34)	Healthy control (34)	SCR
Kienle et al (2018)^117^	Multiple (19)	Healthy control (19)	EEG
Mousa et al (2021)^82^	FND-seizures (17)	Healthy control (20)	Sleep actigraphy
Perez et al (2018)^88^	FND-motor (26)	Healthy control (84)	T_1_-weighted MRI
Pick et al (2020)^89^	FND-mixed (19)	Healthy control (20)	SCL
Stins et al (2015)^100^	FND-unspecified (12)	Healthy control (12)	Postural control
Van der Kruijs et al (2012)^118^	FND-seizures (11)	Healthy control (13)	fMRI
Van der Kruijs et al (2014)^102^	FND-seizures (21)	Healthy control (27)	fMRI
Xue et al (2013)^106^	FND-seizures (15)	Healthy control (15)	EEG

EEG, electroencephalography; fMRI, functional magnetic resonance imaging; MRI, magnetic resonance imaging; SCL, skin conductance levels; SCR, skin conductance response.

#### Structural neuroimaging

Perez and colleagues investigated cortical thickness in people with FND by use of T_1_-weighted structural magnetic resonance imaging to compare participants with FND-motor symptoms and healthy controls.^88^ FND participants with higher SDQ-20 scores (score ≥35; *n* = 10) had significantly reduced cortical thickness in the left caudal (dorsal) anterior cingulate cortex (ACC) relative to controls; this association was not present in the complete FND cohort. Conversely, higher DES depersonalisation/derealisation subscores correlated with increased right lateral occipital cortical thickness in participants with FND.

#### Functional neuroimaging

Van der Kruijs and colleagues used functional magnetic resonance imaging (fMRI) in two studies to investigate alterations in functional connectivity in people with FND-seizures.^102,118^ In the first study,^118^ participants with FND-seizures were compared with healthy controls during four separate fMRI phases, comprising pre- and post-resting states and two cognitive tasks. No significant differences were seen between the FND-seizures group and healthy controls during tasks. However, significant correlations were observed between functional connectivity values and DES, DIS-Q and SDQ-20 scores. Of note, in the FND-seizures cohort functional connectivity was significantly higher within the ACC and inferior frontal gyrus, which also correlated significantly with DES scores.

In a subsequent study, participants with FND-seizures and healthy controls underwent resting-state fMRI.^102^ Relative to controls, those with FND-seizures were observed to have increased co-activation of resting-state networks in frontoparietal (e.g. orbitofrontal cortex), executive (cingulate and insular cortex), sensorimotor (e.g. cingulate gyrus; supplemental motor cortex) and default mode (precuneus, para-cingulate gyri) networks. There was a significant positive correlation between increased functional connectivity in these regions of interest in the FND-seizures group and all dissociation scores (DES, DIS-Q and SDQ-20).

#### Electroencephalography

Two studies employed electroencephalography (EEG) to investigate brain connectivity in FND participants.^106,117^ Xue and colleagues performed EEG with 15 participants with FND-seizures and 15 matched controls; all participants additionally completed the SDQ-20.^106^ The FND-seizures group had less linkage between frontal and posterior brain areas relative to controls; no significant associations between clustering coefficients and SDQ-20 scores were found.

Kienle et al investigated possible cortical indices of emotion regulation in a mixed FND sample and matched controls during an emotion regulation task.^117^ Participants performed one of three emotionally arousing tasks during EEG recording and completed the SDQ-20. This protocol was repeated after a 4 week treatment period of physiotherapy and psychological therapy. Both groups had similar cortical regions of interest in response to unpleasant or neutral stimuli, as well as similar EEG representation during the emotion regulation task (referred to as the ‘regulation effect’). No significant change in these findings was observed after the treatment period; however, a significant association between the regulation effect and SDQ-20 score was seen in the FND group.

#### Skin conductance

Skin conductance is used as a metric of autonomic response to stimuli, employed by two of our included studies.^70,89^ Skin conductance levels (SCL) represent baseline or tonic level of conductance of skin, whereas skin conductance response (SCR) represents a phasic change of skin conductance in response to a stimulus.^119^ Pick and colleagues measured SCL in 17 participants with mixed FND symptoms and matched controls throughout a mirror-gazing dissociation induction protocol.^89^ The FND group had greater average SCL than the controls at all measured points of the protocol; however, this group effect was not significant. No significant associations between dissociation (CADSS score) and SCL were noted.

Herrero et al studied physiological, cognitive and behavioural emotional response to image stimuli in 34 female FND-seizures participants and 34 matched controls.^70^ The DES was used to measure dissociative tendencies. SCR amplitude was significantly lower in the FND-seizures group than in controls in response to all images (*P* = 0.04); however, in response to negative images only there was no significant group difference. A significant negative correlation between DES score and SCR amplitude was observed in the FND-seizures group. A non-significant trend of increased SCR amplitude in response to strong-arousal negative images compared with low-arousal negative images was observed in the FND-seizures group but not in the control group.

#### Sleep actigraphy

Mousa and colleagues investigated objective and subjective sleep complaints in a sample of 17 participants with FND-seizures and 20 age- and gender-matched healthy controls; the DES measured dissociative tendencies.^82^ Participants followed a daily protocol of actigraphy and recording of state dissociative symptoms (using the State Scale of Dissociation^120^), mood, number of FND-seizures and subjective sleep quality. The FND-seizures group reported more disturbed sleep overall than the control group; however, the only significantly worse parameter was sleep quality. Objectively, participants with FND-seizures had significantly worse sleep with respect to efficiency, awakenings and wakefulness after sleep. DES scores were significantly higher in the FND-seizures group relative to the control group. A multivariate linear mixed model did not find any association between sleep parameters (sleep time and number of awakenings) and state dissociation the following day.

#### Postural control

Stins and colleagues investigated postural control in a sample of participants with FND and matched controls.^100^ Participants were asked to stand on a stabilometric platform under various conditions (eyes open, eyes closed and while performing a mental arithmetic task with their eyes open). Physicians administered the CADSS to assess state dissociation. The degree to which participants swayed on the platform under the different conditions was recorded. A greater radius of swaying was noted in the FND group relative to controls; this was more pronounced during the eyes-closed procedure. Distraction using the arithmetic task improved postural stability in the FND group. A significant correlation between dissociative symptoms and postural instability was observed, with higher dissociative scores associated with postural instability during the eyes-closed procedure. However, this was a total sample observation (participants with FND and controls), and this correlation became insignificant when examined in the two groups separately.

### Meta-analysis of dissociative symptom scale scores

#### Somatoform dissociation: FND versus healthy controls

**Fig. 2:**
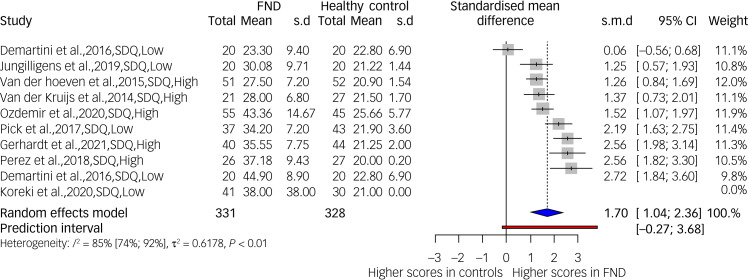
Somatoform dissociation scores in samples with functional neurological disorder (FND) compared with healthy controls. SDQ, Somatoform Dissociation Questionnaire; Low, low risk of bias; High, high risk of bias.

Figure 2 displays results obtained from a meta-analysis comparing SDQ-20 scores of samples with FND and healthy controls (*k* = 9, *n* = 659). A higher score indicates a greater level of somatoform dissociation. The variance between groups was substantial (*I*^2^ = 85.5%), indicating a substantial degree of heterogeneity between the studies. A funnel plot of data available for this meta-analysis is presented in supplementary Fig. 1. It shows asymmetry, which could indicate publication bias. However, this plot also includes a study (Demartini et al^56^) identified in sensitivity analyses as a potential outlier. When this study was removed, the prediction interval shifted to above zero (0.47–3.31, *g* = 1.80, 95% CI 1.25–2.34, *I*^2^ = 75%), although heterogeneity between studies was only reduced to 75%. Supplementary Figs 2 and 3 shows the forest and funnel plots when Demartini et al is removed. In the original meta-analysis, all studies were case–control, five were at high risk of bias and four were at low risk of bias (Demartini et al gave data for participants with motor symptoms and seizures). A meta-regression showed that the studies’ risk of bias category is not a significant effect size predictor (*P* = 0.50). All studies included in all meta-analyses were case–control studies because of our data inclusion principles outlined in the Methods section above.

#### Psychoform dissociation: all samples with FND versus healthy, neurological and psychiatric controls

**Fig. 3:**
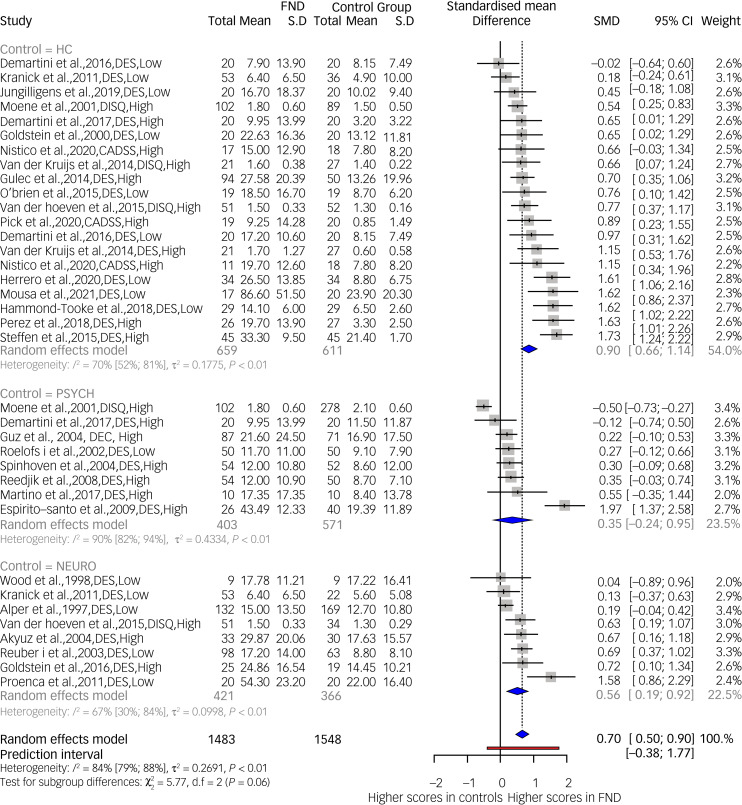
Psychoform dissociation scores in samples with functional neurological disorder (FND) compared with healthy (HC), psychiatric (PSYCH) and neurological (NEURO) controls. Low, low risk of bias; High, high risk of bias.

Figure 3 presents results of the mixed-effects model meta-analysis investigating dissociative symptom scale scores (combined psychoform dissociation measure) in all samples with FND (FND-seizures, FND-motor, FND-sensory, mixed) compared with healthy, psychiatric or neurological controls (*k* = 36, *n* = 3031). Overall, the test between control subgroups was not significant (*Q*(2) = 5.77, *P* = 0.056). The FND groups showed a significant effect towards increasing psychoform dissociation compared with healthy controls (*g* = 0.90, 95% CI 0.66–1.14, *I*^2^ = 70%) and neurological controls (*g* = 0.56, 95% CI 0.19–0.92, *I*^2^ = 67%). Psychiatric controls also showed an effect towards lower dissociation scores compared with the FND groups but heterogeneity was very high (*g* = 0.35, 95% CI 0.24–0.95, *I^2^* = 90%), suggesting that, across studies, the psychiatric samples were too dissimilar for interpretation at this stage. A funnel plot of data available for this meta-analysis is given in supplementary Fig. 4. It shows some smaller studies with large effect sizes, which might indicate some publication bias. All studies in this analysis were case–control, 21 data comparisons were from studies with high risk of bias, and the final 15 were from studies with low risk of bias. A meta-regression showed no significant effect of the studies’ risk of bias category (*P* = 0.97).

#### Psychoform dissociation: FND subgroups (FND-seizures, FND-motor) versus healthy controls

**Fig. 4:**
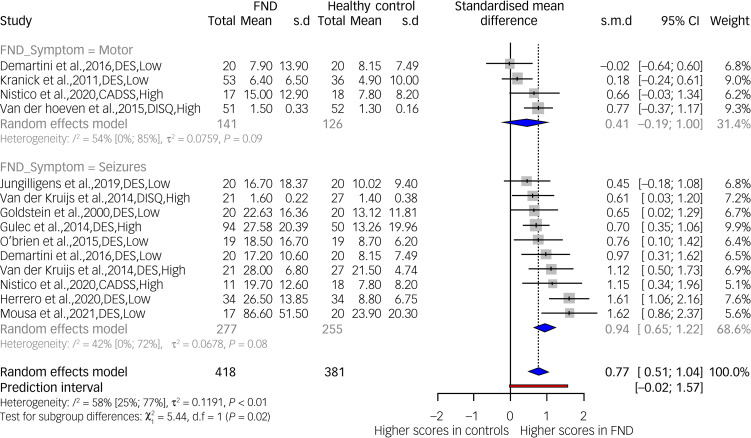
Psychoform dissociation scores in samples with seizure symptoms and motor symptoms of functional neurological disorder (FND) compared with healthy controls. Low, low risk of bias; High, high risk of bias.

Figure 4 presents data from the mixed-effects model meta-analysis for the subgroups FND-seizures and FND-motor (*k* = 14, *n* = 799). Four valid data comparisons were available for healthy controls and FND-motor samples and ten for FND-seizures. The FND-seizures group showed a significant effect of increasing psychoform dissociation compared with the FND-motor group (*Q*(1) = 5.44, *P* = 0.020). As there were only four data points available for the FND-motor group, compared with ten for FND-seizures group, we checked results assuming common heterogeneity (*τ*^2^ = 0.071) and the effect remained significant (*P* = 0.020).

The effect of FND-seizures on increasing psychoform dissociation was significant compared with healthy controls, with a large effect size (*g* = 0.94, 95% CI 0.65–1.22). There was also an effect towards greater dissociation scores in the FND-motor group compared with healthy controls; however, this was a smaller effect compared with that for FND-seizures (*g* = 0.40, 95% CI −0.18 to 1.00). Estimates of between-study heterogeneity ranged from 53.7% for FND-motor to 42% for FND-seizures and was 58.3% overall; therefore, we can be confident that each group represents the target population. A funnel plot of data available for this meta-analysis is given in supplementary Fig. 5. As above, it shows a study with a large effect size, despite large standard error.^82^ All studies included were case–control. Data comparisons were available from six studies at high risk of bias and eight at low risk of bias. A meta-regression showed no significant effect of the studies’ risk of bias category (*P* = 0.81).

## Discussion

This review presents evidence that dissociation is an important feature of FND. In relation to the three aims of this study, our principal findings are as follows. First, that FND frequently involves comorbid dissociative disorders. Second, psychoform and somatoform dissociative symptoms are commonly present in FND and appear to vary with FND presentation. Third, we found several potential biological and clinical associations of dissociation in FND that merit further exploration.

### Dissociative disorders are common comorbidities in FND

Fewer studies than expected commented on comorbid dissociative disorders in FND samples. In the nine studies that did, the proportion of participants with FND found to have comorbid dissociative disorder ranged considerably, from 8% to 80%.^14,41–48^ The most common dissociative disorder diagnosed was dissociative disorder not otherwise specified (DDNOS) – a subtype previously highlighted as being the most prevalent in epidemiological studies.^121,122^ When compared with prevalence rates reported for the general population, dissociative disorder rates are higher in FND groups. A review of dissociative disorder prevalence rates in community and clinical samples suggested an overall lifetime prevalence of 10%.^123^ Similarly, a recent meta-analysis of college student populations (*n* = 31 905) reported an overall prevalence of 11%.^124^ Prevalence of dissociative disorder might be higher in clinical populations; for example, Foote and colleagues observed an overall prevalence of 29% in 82 consecutive out-patient psychiatric clinic attendees, and Ross et al observed considerably higher lifetime prevalence in in-patients (28–44.5%).^125,126^ A separate study found that general psychiatric in-patients were more likely to have a comorbid dissociative disorder if they scored above 30 on the DES, with patients in this bracket having a prevalence of up to 80%.^127^ The majority of studies reviewed here reported prevalence rates within the clinical range observed by Ross et al, and in some cases considerably higher. However, two of the included studies^42,45^ had prevalence rates of 10% or lower, commensurate with the prevalence rate of dissociative disorders in the general population.^123,124^

The prevalence of dissociative disorders seen in the samples with FND presented in this review supports the notion that FND and dissociative disorders might share similar mechanisms and aetiology (e.g. traumatic experiences, chronic/severe stressors), in addition to symptoms, supporting the classification of FND as a dissociative disorder in the ICD.^2,4^ Given that dissociative disorders are prevalent in other psychiatric populations,^125,126^ dissociation as an explanatory mechanism for other mental disorders merits exploration. For FND, this proximity invites questions about treatment options and whether some people with FND would benefit from treatments that target dissociative disorders and symptoms, including those currently being evaluated in FND such as eye movement desensitisation and reprocessing (EMDR)^128–131^ and dynamic psychotherapies.^132,133^

### Dissociative symptoms are elevated in FND and vary by subgroup

Dissociative symptoms, as measured through validated scales, ranged widely in the FND samples. However, most studies reported elevated dissociative symptom scores in FND samples that were above clinical cut-off scores and/or the scores reported for comparison groups. Elevated scores were observed for both psychoform and somatoform manifestations of dissociation.

The DES was the most used measure of psychoform dissociation. DES scores have recently been meta-analysed in psychiatric populations.^3^ In that meta-analysis, the mean DES score for FND was reported at 25.6, based on 20 studies, and dissociative disorders had a mean score of 38.9;^3^ 11 studies from our review reported mean or median scores higher than this, and 4 reported scores above the mean score for dissociative disorders. We found 11 studies that used other psychoform dissociation scales (e.g. CADSS, MDI, CDS) – the majority of FND groups scored higher on these dissociative measures when compared with controls.

In our subgroup meta-analysis investigating psychoform dissociation in FND groups versus different control groups, a large effect towards higher dissociation scores was found in the FND groups compared with healthy controls. This effect was also observed in the comparison with neurological controls, but of a medium effect size. High heterogeneity prevents us from confirming an effect between FND groups and psychiatric controls. The last finding may have been affected by the amalgamation of multiple psychiatric populations into one group. Four out of eight studies included a mixed psychiatric control group, while the remaining four involved somatisation, eating disorder, chronic pain and depression.

The SDQ-20 was the next most used dissociative symptom scale, and the only ‘pure’ measure of somatoform dissociation. Mean SDQ-20 scores in samples with dissociative disorder have been reviewed previously, observing mean scores >30 for FND samples, >43 for DDNOS and DPDR samples and >50 for DID samples.^134^ Using these as a reference, FND samples (of a total of 25) endorsed mean or median SDQ-20 scores >30, supporting the view that compartmentalisation is a significant feature of FND. Our meta-analysis comparing SDQ scores in people with FND and healthy controls displayed high heterogeneity between studies (Fig. 2); however, the prediction interval shows that research in this area has generally found people with FND to exhibit higher somatoform dissociation than healthy controls. Only the comparison from Demartini et al^56^ involving participants with FND-seizures compared with healthy controls showed a non-significant effect. Sensitivity analyses suggested that this study was an outlier, and removing it shifted the prediction interval to greater than zero, indicating that future studies are likely to find the same pattern of results. Despite this, large heterogeneity remained (75%), meaning it is likely that the FND samples contained important subgroup populations that would need to be studied before effect size estimates could be interpreted. However, subgroup analysis was not possible as there were fewer than ten studies.

Our results suggested that there might be different dissociative symptom profiles across FND subgroups, specifically elevated psychoform dissociation in FND-seizures and greater somatoform dissociation in FND-motor samples. Guz and colleagues,^66^ for example, reported the highest DES scores in an FND-seizures sample (mean score 27) relative to FND-motor (mean 12) and FND-sensory samples (mean 7). Nearly all of the FND-motor sample DES scores were comparable to the aforementioned mean scores of non-clinical populations, or substantially lower.^135^ Only in Tezcan and colleagues’ study did an FND-motor group exhibit high psychoform dissociation (mean DES = 25).^48^ Demartini and colleagues directly compared FND-seizures and FND-motor groups and noted an inverse relationship between DES and SDQ-20 scores in the two groups (FND-seizures mean DES = 17.2, mean SDQ-20 = 23.3; FND-motor mean DES = 7.9, mean SDQ-20 = 44.9).^56^ This observation was not totally consistent; some FND-seizures groups scored highly on the SDQ-20 (median SDQ-20 = 34 in Pick et al^90^; mean SDQ-20 = 38 in Koreki et al^76^), with no clear methodological differences that could account for this.

Significantly higher SDQ-20 scores in an FND-seizures sample relative to epilepsy controls were reported in a study in which the DES and CDS were unable to differentiate participants with FND from controls.^136^ The authors argued therefore that compartmentalisation symptoms are more characteristic of FND-seizures, whereas detachment symptoms are less prominent. In our review, the highest DIS-Q score was also in an FND-seizures group.^85^ Perhaps, therefore, these results indicate that people with the FND-seizures subtype have a greater overall tendency to dissociate in general, with respect to both somatoform and psychoform symptoms. The discrepant findings noted here require further examination in additional studies using measures of both somatoform and psychoform dissociation in FND-seizures and FND-motor samples.

A potential explanation of the observed trends is that FND motor symptoms are indeed comparable to so-called somatoform dissociative symptoms, whereas psychoform dissociative symptoms are more common in people with FND-seizures. Indeed, the SDQ-20 contains several items that map directly onto FND motor symptoms, whereas there is only one item regarding attacks resembling seizures. One of the developers of the SDQ-20 has already called for physical ‘conversion’ symptoms to be relabelled as somatoform dissociative symptoms.^134^

Several limitations affect the generalisability of the observations outlined above. The majority of studies that addressed FND subtypes focused on FND-seizures; very few studies specified the FND subtype and most involved people with FND endorsing multiple symptoms. There were only three studies presenting FND-sensory or ‘mixed’ (F44.6 and F44.7 respectively) subtype specific scores^48,66,97^ and our subgroup meta-analysis indicated that FND-seizures groups exhibited different levels of psychoform dissociation from FND-motor groups. This meta-analysis indicates the need for more FND-motor and healthy control comparisons. Importantly, future studies should examine closely the effect on SDQ-20 total scores of items pertaining to the particular FND symptoms experienced by each respondent. One methodological solution is to rerun analyses excluding items that resemble FND symptoms common in the sample under investigation.

Although the DES and SDQ-20 scales were the most commonly used dissociation scales, most studies did not administer both and thus did not screen for both types of dissociative symptoms. It has been suggested that rather than existing as a general attribute, dissociation represents a diverse and not necessarily connected cluster of symptoms; consequently, scales such as the MDI, which measure specific psychoform dissociative symptoms such as depersonalisation and identity dissociation, might be more valuable for assessing dissociative symptom profiles.^137^ Reporting bias, and additionally recall bias, are also potential limitations of self-report scales, which may be mitigated by use of scales that are clinician-administered and/or state-based, such as the CADSS.

The large values for measures of dispersion seen across the studies indicate that although some individuals with FND report elevated dissociative tendencies, there are some who appear less affected by dissociation. If specific FND presentations present with different types of dissociative symptoms, as suggested by our results, then omission of measures of psychoform and/or somatoform dissociation might result in falsely low or unrepresentative results. However, another interpretation is that dissociative symptoms, although common, are not a ubiquitous feature of FND. More studies that administer both psychoform and somatoform dissociative scales to people with various FND subtypes will help to establish more concretely whether the observed trends represent the population.

### Biological associations of dissociation in FND

Little is known about the biological processes underlying pathological dissociation. Roydeva & Reinders^15^ recently systematically reviewed studies exploring potential biomarkers associated with pathological dissociation transdiagnostically, including dissociative disorders, FND, and affective, personality and stress-related disorders.^15^ Increased neural activity was observed in several brain regions in the dissociative groups relative to healthy or clinical control groups, in both resting-state and task-based functional neuroimaging studies. This was consistent in regions of the prefrontal cortex, insula and ACC – findings also reported by Drane et al in a previous review^138^ and in models of dissociation in specific disorders, including PTSD and DID.^139,140^

Trends in connectivity alterations included increased connectivity from amygdala seed regions to prefrontal cortex (e.g. dorsolateral, medial, orbitofrontal), precuneus and superior parietal cortex. In addition, structural imaging studies found evidence of volumetric reductions in several regions in the dissociative groups, including the hippocampus, thalamus and basal ganglia. Other trends included a negative correlation between tumour necrosis factor alpha and dissociative symptoms, and a positive correlation with interleukin 6, raising questions about an interaction between inflammation and dissociative symptoms;^15^ however, this process may be mediated by other comorbid disorders, such as depression.^141^ Further research is needed to better understand the neurobiological basis of dissociative symptoms and disorders.

Our review found a noticeably smaller number of studies examining the relationships between biological factors and dissociative symptoms in people with FND when compared with the Roydeva & Reinders review.^15^ This is likely due to differing study inclusion criteria; we specified that our studies describe dissociative symptoms or comorbid disorders with validated measures in people with FND, whereas the aforementioned review accepted diagnosis of FND alone as a marker of dissociation. Despite this disparity, there were some common trends noted: three of the identified studies from our review shared consistent findings with some of the reports of altered neural circuitry described above.^88,102,118^ Volumetric and functional connectivity changes were observed within several brain regions, including the ACC – a region implicated in action planning, decision-making and empathy-related responses. The ACC is consistently highlighted as an area of interest in FND studies.^138,142,143^ Increased functional connectivity and neural activity within this cortical region, among several other related regions described earlier, has been advocated as a potential biomarker for pathological dissociation and for FND.^138,144^ Whether these demonstrable alterations in neural circuitry can be translated into an *in vivo* biomarker of disorder severity or response to treatment remains to be explored. Studies applying this method have shared promising results.^145,146^

Two relevant studies from Labate and colleagues could not be included in this review as they did not meet our inclusion criteria.^147,148^ The first reported on cortical thinning in similar brain regions, using voxel-based morphometry and cortical thickness MRI techniques.^147^ Significant loss of grey matter volume in participants with FND-seizures was observed in primary motor and premotor cortices, the supplementary motor area and the ACC.^147^ Thickness alterations in these regions of interest were not associated with dissociation (on DES and SDQ scores); however, SDQ scores were significantly negatively associated with reduced cortical thickness in the left inferior frontal gyrus and the left central sulcus.^147^ The second, more recent, study contrasted similar brain regions of interest in subgroups of healthy participants and participants with depression and FND-seizures.^148^ Relative to healthy controls, both the depression and FND-seizures subgroups had noticeable thinning of the ACC and lateral orbitofrontal cortext on MRI. Although this was more pronounced in participants with depression, a significant positive relationship between SDQ scores and morphological changes in the right medial orbitofrontal cortext was observed.^148^

Other biological findings were more variable. Two studies measured skin conductance levels in people with FND, with inconsistent results.^70,89^ These inconsistent findings are similar to those previously reported in Drane et al's review.^138^ One possible explanation is that in FND, interoceptive impairments lead to a discordance between subjective and autonomic responses. This is reflected further in other studies that have reported impaired interoceptive accuracy of people with FND.^56,57,76,89^ Owing to the limited number of studies found and the variety of objective measures, a meta-analysis was not possible for the biological associations of dissociation in FND.

### Clinical features associated with dissociation in FND

Several studies in this review offer compelling evidence in support of using dissociative tendencies, symptoms or comorbidity as a potential prognostic marker in FND. Many of these studies pertained to the FND-seizures subtype. Severity or frequency of seizure symptoms, and presence of ongoing seizures relative to remission states, were seen in people with FND who reported greater degrees of dissociative symptoms.^51,78,80,87,93,103^ Quality of life in people with FND-seizures was also reportedly affected by degree of dissociation, even after controlling for a number of other factors.^81,115^ Other studies observed varying severity of presentation with level of dissociation in general FND populations. The concerning link between dissociative symptoms and suicide has been established previously in psychiatric samples.^26,68,149^ More longitudinal studies could therefore be helpful to further establish the link between dissociative symptoms and FND severity or quality of life.

Several studies observed that alexithymia and emotion dysregulation were associated with dissociation severity in FND participants.^53,55,75^ Number of FND symptoms, earlier onset of symptoms and likelihood of symptom severity requiring in-patient management were also predicted, to a degree, by dissociative symptoms.^14,45,46^ Other findings included elevated general psychopathology and psychiatric comorbidity in FND participants with greater dissociative tendencies.^14,101^

Taken together, these findings have important clinical implications. Dissociation scales might provide a novel means of categorising FND severity, prognosis and guiding treatment. Reflecting this notion, some of the studies in this review adopted the SDQ-20 specifically as a marker of FND severity.^75,99^ It might be beneficial to measure psychoform or somatoform dissociative symptoms as an outcome for FND in future studies. In addition, high scores on dissociation measures in people with FND also reporting previous trauma might indicate the presence of post-traumatic symptoms,^90^ which would need to be addressed specifically in any effective clinical intervention. The associations between traumatic experiences, dissociative and post-traumatic symptoms in FND are important and understudied; although this unfortunately goes beyond the scope of this paper, it merits further exploration in future studies.

### Strengths and limitations of the review

Our review has several strengths. First, we have comprehensively summarised existing data on both dissociative disorders and dissociative symptoms across different FND symptom types, and our subgroup analyses have highlighted potentially important subgroup differences. We also included studies that used a range of validated dissociation measures, ensuring that our review captured as much of the existing data as possible and providing insights into the relative prevalence of different types of dissociative symptoms (e.g. detachment, compartmentalisation) in the FND samples. Our findings have also indicated several important directions for future research on biological mechanisms and clinical implications of dissociation in FND.

We acknowledge some limitations in the methods used. Regarding the search strategy, terms for cognitive FND symptoms were not included because we designed the search strategy in 2019, before these symptoms were formally classified as FND in ICD-11. Overall, the meta-analyses indicated a general problem of large heterogeneity between studies, which limits the certainty with which some conclusions can be drawn. This is especially evident in the neurological and psychiatric control groups, which each subsumed several different clinical disorders. In addition to this, funnel plots all showed asymmetry within the data. There are cases of small studies reporting a large effect size and large standard error, which is a potential indictor of publication bias. However, this could also be reflective of the high heterogeneity and the presence of outliers. We were also unable to explore the influence of associated risk factors such as trauma on dissociation scores in the meta-analysis, which might be of value in future. Generally, there were only a small number of studies that investigated the relationship between dissociation scores and biological measures, with inconsistent findings on several measures. Finally, although we excluded any study that stated that participants with FND had comorbid neurological disorders, not all studies explicitly declared this (or that comorbid neurological disorders were excluded), and therefore there is a possibility that some of the data included may have not been FND-specific.

### Future directions and clinical implications

The findings presented here indicate that dissociation is a pervasive and clinically relevant feature in FND, also lending support to the view that dissociation is a possible underlying mechanism. Further research is needed to examine more rigorously the role of dissociative processes in the generation of FND symptoms, with methods designed to examine causal hypotheses. Improved understanding of the role of dissociation in FND will have implications for diagnosis and classification, with potential to facilitate resolution of the discrepancy between the ICD-11 and DSM-5 classifications.

Despite the considerable literature reviewed here, few studies investigated the potentially differing degrees of psychoform and somatoform dissociation in different FND subtypes. Furthermore, few studies explicitly sought to examine the distinction between compartmentalisation and detachment subtypes of dissociation in FND subgroups. More studies should closely examine these different forms of dissociation, their underlying aetiological and neurobiological basis, and how they relate to the occurrence of core FND symptoms such as seizure, motor, cognitive and sensory symptoms.

From a clinical perspective, this review highlighted several possible implications. To accurately capture all of the symptomatology and experiences of patients with FND, clinicians might consider screening for both somatoform and psychoform dissociative symptoms, as they are evidently overexpressed in this population and are associated with adverse clinical features and outcomes. For individuals with FND who report significant dissociation, a dedicated clinical interview to assess possible comorbid dissociative disorders would be another consideration, as would be a more detailed screening for past trauma and possible post-traumatic symptoms.

A key research question is whether level of dissociative symptoms could constitute a prognostic indicator of FND severity. Lastly, there are some appreciable potential biomarkers of dissociation that, if explored further, could aid in understanding how this process occurs and potentially act as guides to monitoring treatment efficacy and/or developing novel treatments for the disorder.

## Data Availability

Data availability is not applicable to this article as no new data were created or analysed in this study.

## Supplementary material

For supplementary material accompanying this paper visit http://doi.org/10.1192/bjo.2022.597. click here to view supplementary material

## Appendix

Dissociation and functional neurological disorder terms used in the database search. The categories ‘dissociation’ and ‘functional neurological disorder’ were combined with ‘AND’; the list of terms within each category were combined with ‘OR’:

**Dissociation terms**

Dissociative

Dissociation

Depersonali*tion

Dereali*ation

Detachment

Compartmentali*ation

**Functional neurological disorder terms**

Functional neurological

Functional motor

Functional weakness

Conversion disorder

Psychogenic

Non*epileptic

Dissociative seizure

Hysteri*

## References

[1] American Psychiatric Association. Diagnostic and Statistical Manual of Mental Disorders (5th edn) (DSM-5). American Psychiatric Association, 2013.

[2] World Health Organization. ICD-11: International Classification of Diseases 11th Revision . WHO, 2019.

[3] Lyssenko L, Schmahl C, Bockhacker L, Vonderlin R, Bohus M, Kleindienst N. Dissociation in psychiatric disorders: a meta-analysis of studies using the Dissociative Experiences Scale. Am J Psychiatry 2018; 175: 37–46. 28946763 10.1176/appi.ajp.2017.17010025

[4] Brown RJ, Cardeña E, Nijenhuis E, Sar V, van der Hart O. Should conversion disorder be reclassified as a dissociative disorder in DSM–V? Psychosomatics 2007; 48: 369–78. 17878494 10.1176/appi.psy.48.5.369

[5] Brown RJ, Reuber M. Psychological and psychiatric aspects of psychogenic non-epileptic seizures (PNES): a systematic review. Clin Psychol Rev 2016; 45: 157–82. 27084446 10.1016/j.cpr.2016.01.003

[6] Cardeña E, Carlson E. Acute stress disorder revisited. Annu Rev Clin Psychol 2011; 7: 245–67. 21275643 10.1146/annurev-clinpsy-032210-104502

[7] Dell PF. Dissociative phenomenology of dissociative identity disorder. J Nerv Ment Dis 2002; 190: 10–5. 11838024 10.1097/00005053-200201000-00003

[8] Medford N, Baker D, Hunter E, Sierra M, Lawrence E, Phillips ML, Chronic depersonalization following illicit drug use: a controlled analysis of 40 cases. Addiction 2003; 98: 1731–6. 14651505 10.1111/j.1360-0443.2003.00548.x

[9] Romero-López MJ. A review of the dissociative disorders: from multiple personality disorder to the posttraumatic stress. Anales de Psicol 2016; 32: 448–56.

[10] Hunter E, Phillips ML, Chalder T, Sierra M, David AS. Depersonalisation disorder: a cognitive–behavioural conceptualisation. Behav Res Ther 2003; 41: 1451–67. 14583413 10.1016/s0005-7967(03)00066-4

[11] Ross CA, Joshi S, Currie R. Dissociative experiences in the general population. Am J Psychiatry 1990; 147: 1547–52. 2221172 10.1176/ajp.147.11.1547

[12] Hendrickson R, Popescu A, Ghearing G, Bagic A. Thoughts, emotions, and dissociative features differentiate patients with epilepsy from patients with psychogenic nonepileptic spells (PNESs). Epilepsy Behav 2015; 51: 158–62. 26283304 10.1016/j.yebeh.2015.07.016

[13] Spiegel D. Dissociation in the DSM5. J Trauma Dissociation 2010; 11: 261–5. 20603761 10.1080/15299731003780788

[14] Yayla S, Bakım B, Tankaya O, Ozer OA, Karamustafalioglu O, Ertekin H, Psychiatric comorbidity in patients with conversion disorder and prevalence of dissociative symptoms. J Trauma Dissociation 2015; 16: 29–38. 25365395 10.1080/15299732.2014.938214

[15] Roydeva MI, Reinders AA. Biomarkers of pathological dissociation: a systematic review. Neurosci Biobehav Rev 2021; 123: 120–202. 33271160 10.1016/j.neubiorev.2020.11.019

[16] Treise C, Perez J. The role of dissociative compartmentalization in difficult-to-treat psychotic phenomena. Front Psychol 2021; 12: 1093. 10.3389/fpsyg.2021.533884 PMC8062699 33897507

[17] Cardeña E, Gušić S, Cervin M. A network analysis to identify associations between PTSD and dissociation among teenagers. J Trauma Dissociation [Epub ahead of print] 22 Oct 2021. Available from: 10.1080/15299732.2021.1989122. 34678139

[18] Schimmenti A, Sar V. A correlation network analysis of dissociative experiences. J Trauma Dissociation 2019; 20: 402–19. 30714885 10.1080/15299732.2019.1572045

[19] Holmes EA, Brown RJ, Mansell W, Fearon RP, Hunter EC, Frasquilho F, Are there two qualitatively distinct forms of dissociation? A review and some clinical implications. Clin Psychol Rev 2005; 25(1): 1–23. 15596078 10.1016/j.cpr.2004.08.006

[20] Nijenhuis ER, Spinhoven P, Van Dyck R, Van der Hart O, Vanderlinden J. The development and psychometric characteristics of the Somatoform Dissociation Questionnaire (SDQ-20). J Nerv Ment Dis 1996; 184: 688–94. 8955682 10.1097/00005053-199611000-00006

[21] van der Boom KJ, van den Hout MA, Huntjens RJC. Psychoform and somatoform dissociation, traumatic experiences, and fantasy proneness in somatoform disorders. Pers Individ Dif 2010; 48: 447–51.

[22] Bernstein EM. Development, Reliability and Validity of a Dissociation Scale (Derealization, Depersonalization, Test Construction). American University, 1986.

[23] Hansen M, Ross J, Armour C. Evidence of the dissociative PTSD subtype: a systematic literature review of latent and class and profile analytic studies of PTSD. J Affect Disord 2017; 213: 59–69. 28192736 10.1016/j.jad.2017.02.004

[24] Burton MS, Feeny NC, Connell AM, Zoellner LA. Exploring evidence of a dissociative subtype in PTSD: baseline symptom structure, etiology, and treatment efficacy for those who dissociate. J Consult Clin Psychol 2018; 86: 439–51. 29683701 10.1037/ccp0000297 PMC5918299

[25] Huijstee JV, Vermetten E. The dissociative subtype of post-traumatic stress disorder: research update on clinical and neurobiological features. Behav Neurobiol PTSD 2017; 38: 229–48. 10.1007/7854_2017_33 29063485

[26] Calati R, Bensassi I, Courtet P. The link between dissociation and both suicide attempts and non-suicidal self-injury: meta-analyses. Psychiatry Res 2017; 251: 103–14. 28196773 10.1016/j.psychres.2017.01.035

[27] Langeland W, Jepsen EKK, Brand BL, Kleven L, Loewenstein RJ, Putnam FW, The economic burden of dissociative disorders: a qualitative systematic review of empirical studies. Psychol Trauma 2020; 12(7): 730–8. 32212775 10.1037/tra0000556

[28] Brière J. MDI, Multiscale Dissociation Inventory: Professional Manual. Psychological Assessment Resources, 2002.

[29] Bremner JD, Krystal JH, Putnam FW, Southwick SM, Marmar C, Charney DS, Measurement of dissociative states with the Clinician-Administered Dissociative States Scale (CADSS). J Trauma Stress 1998; 11: 125–36. 9479681 10.1023/A:1024465317902

[30] Vanderlinden J, Van Dyck R, Vandereycken W, Vertommen H, Verkes RJ The Dissociation Questionnaire (DIS-Q): development and characteristics of a new self-report questionnaire. Clin Psychol Psychother 1993; 1: 21–7.

[31] Steinberg M, Rounsaville B, Cicchetti DV. The Structured Clinical Interview for DSM-III-R Dissociative Disorders: preliminary report on a new diagnostic instrument. Am J Psychiatry 1990; 147: 76–82. 2293792 10.1176/ajp.147.1.76

[32] Moskalewicz A, Oremus M. No clear choice between Newcastle–Ottawa scale and appraisal tool for cross-sectional studies to assess methodological quality in cross-sectional studies of health-related quality of life and breast cancer. J Clin Epidemiol 2020; 120: 94–103. 31866469 10.1016/j.jclinepi.2019.12.013

[33] Higgins JPT, Li T, Deeks J. Obtaining standard errors from confidence intervals and P values: absolute (difference) measures. In Cochrane Handbook for Systematic Reviews of Interventions, *version 6.3* (updated February 2022) (eds JPT Higgins, J Thomas, J Chandler, M Cumpston, T Li, MJ Page, VA Welch): Ch. 6.3.1. Cochrane, 2022 (https://training.cochrane.org/handbook/current/chapter-06#section-6-3-1).

[34] Viechtbauer W. Bias and efficiency of meta-analytic variance estimators in the random-effects model. J Educ Behav Stat 2005; 30: 261–93.

[35] Hartung J, Knapp G. An alternative test procedure for meta-analysis. In Meta-analysis: New Developments and Applications in Medical and Social Sciences (eds R Schulze, H Holling, D Böhning): 53–69. Hogrefe & Huber Publishers, 2003.

[36] Balduzzi S, Rücker G, Schwarzer G. How to perform a meta-analysis with R: a practical tutorial. Evid Based Ment Health 2019; 22: 153–60. 31563865 10.1136/ebmental-2019-300117 PMC10231495

[37] Harrer M, Cuijpers P, Furukawa T, Ebert D Doing Meta-Analysis With R: A Hands-On Guide. CRC Press, 2021.

[38] Cochran WG. The combination of estimates from different experiments. Biometrics 1954; 10: 101–29.

[39] Higgins JP, Thompson SG. Quantifying heterogeneity in a meta-analysis. Stat Med 2002; 21: 1539–58. 12111919 10.1002/sim.1186

[40] Wells GA, Shea B, O'Connell D, Peterson J, Welch V, Losos M, The Newcastle–Ottawa Scale (NOS) for Assessing the Quality of Nonrandomised Studies in Meta-Analyses. The Ottawa Hospital Research Institute, 2000 (https://www.ohri.ca//programs/clinical_epidemiology/oxford.asp).

[41] Litwin R, Cardeña E. Demographic and seizure variables, but not hypnotizability or dissociation, differentiated psychogenic from organic seizures. J Trauma Dissociation 2001; 1: 99–122.

[42] Marchetti RL, Kurcgant D, Neto JG, Von Bismark MA, Fiore LA. Evaluating patients with suspected nonepileptic psychogenic seizures. J Neuropsychiatry Clin Neurosci 2009; 21: 292–8. 19776309 10.1176/jnp.2009.21.3.292

[43] Akyüz F, Gökalp PG, Erdiman S, Oflaz S, Karşidağ Ç. Conversion disorder comorbidity and childhood trauma. Noro Psikiyatr Ars 2017; 54: 15–20. 28566953 10.5152/npa.2017.19184 PMC5439465

[44] Baillés E, Pintor L, Fernandez-Egea E, Torres X, Matrai S, De Pablo J, Psychiatric disorders, trauma, and MMPI profile in a Spanish sample of nonepileptic seizure patients. Gen Hosp Psychiatry 2004; 26: 310–5. 15234827 10.1016/j.genhosppsych.2004.04.003

[45] Moene FC, Spinhoven P, Hoogduin K, Sandyck P, Roelofs K Hypnotizability, dissociation and trauma in patients with a conversion disorder: an exploratory study. Clin Psychol Psychother 2001; 8: 400–10.

[46] Roelofs K, Hoogduin KA, Keijsers GP, Näring GW, Moene FC, Sandijck P. Hypnotic susceptibility in patients with conversion disorder. J Abnorm Psychol 2002; 111: 390–5. 12003460 10.1037//0021-843x.111.2.390

[47] Scévola L, Teitelbaum J, Oddo S, Centurión E, Loidl CF, Kochen S, Psychiatric disorders in patients with psychogenic nonepileptic seizures and drug-resistant epilepsy: a study of an Argentine population. Epilepsy Behav 2013; 29: 155–60. 23969203 10.1016/j.yebeh.2013.07.012

[48] Tezcan E, Atmaca M, Kuloglu M, Gecici O, Buyukbayram A, Tutkun H. Dissociative disorders in Turkish inpatients with conversion disorder. Compr Psychiatry 2003; 44: 324–30. 12923711 10.1016/S0010-440X(03)00087-7

[49] Akyuz G, Kugu N, Akyuz A, Dogan O. Dissociation and childhood abuse history in epileptic and pseudoseizure patients. Epileptic Disord 2004; 6: 187–92. 15544989

[50] Alper K, Devinsky O, Perrine K, Luciano D, Vazquez B, Pacia S, Dissociation in epilepsy and conversion nonepileptic seizures. Epilepsia 1997; 38: 991–7. 9579937 10.1111/j.1528-1157.1997.tb01481.x

[51] Bodde NM, Janssen AM, Theuns C, Vanhoutvin JF, Boon PA, Aldenkamp AP. Factors involved in the long-term prognosis of psychogenic nonepileptic seizures. J Psychosom Res 2007; 62: 545–51. 17467409 10.1016/j.jpsychores.2006.11.015

[52] Boesten N, Myers L, Wijnen B. Quality of life and psychological dysfunction in traumatized and nontraumatized patients with psychogenic nonepileptic seizures (PNES). Epilepsy Behav 2019; 92: 341–4. 30769279 10.1016/j.yebeh.2019.01.024

[53] Brown RJ, Bouska JF, Frow A, Kirkby A, Baker GA, Kemp S, Emotional dysregulation, alexithymia, and attachment in psychogenic nonepileptic seizures. Epilepsy Behav 2013; 29: 178–83. 23973643 10.1016/j.yebeh.2013.07.019

[54] Cope SR, Smith JG, King T, Agrawal N. Evaluation of a pilot innovative cognitive-behavioral therapy-based psychoeducation group treatment for functional non-epileptic attacks. Epilepsy Behav 2017; 70(Pt A): 238–44. 28454061 10.1016/j.yebeh.2017.02.014

[55] Del Río-Casanova L, González-Vázquez AI, Justo A, Andrade V, Páramo M, Brenlla J, The role of emotion dysregulation in conversion disorder. Act Esp Psiquiatr 2018; 46: 92–103. 29892968

[56] Demartini B, Goeta D, Barbieri V, Ricciardi L, Canevini MP, Turner K, Psychogenic non-epileptic seizures and functional motor symptoms: a common phenomenology? J Neurol Sci 2016; 368: 49–54. 27538601 10.1016/j.jns.2016.06.045

[57] Demartini B, Goeta D, Romito L, Anselmetti S, Bertelli S, D'Agostino A, Anorexia nervosa and functional motor symptoms: two faces of the same coin? J Neuropsychiatry Clin Neurosci 2017; 29: 383–90. 28558480 10.1176/appi.neuropsych.16080156

[58] Ekanayake V, Kranick S, LaFaver K, Naz A, Frank Webb A, LaFrance WC Jr, Personality traits in psychogenic nonepileptic seizures (PNES) and psychogenic movement disorder (PMD): neuroticism and perfectionism. J Psychosom Res 2017; 97: 23–9. 28606495 10.1016/j.jpsychores.2017.03.018 PMC5572831

[59] Espirito-Santo HMA, Pio-Abreu JL. Dissociative disorders and other psychopathological groups: exploring the differences through the somatoform dissociation questionnaire (SDQ-20). Braz J Psychiatry 2007; 29: 354–8. 17713705 10.1590/s1516-44462006005000039

[60] Espirito-Santo H, Pio-Abreu JL. Psychiatric symptoms and dissociation in conversion, somatization and dissociative disorders. Aust N Z J Psychiatry 2009; 43: 270–6. 19221916 10.1080/00048670802653307

[61] Evren C, Can S. Clinical correlates of dissociative tendencies in male soldiers with conversion disorder. Isr J Psychiatry Relat Sci 2007; 44: 33. 17665809

[62] Gerhardt C, Hamouda K, Irorutola F, Rose M, Hinkelmann K, Buchheim A, Insecure and unresolved/disorganized attachment in patients with psychogenic nonepileptic seizures. J Acad Consult Liaison Psychiatry 2021; 62: 337–44. 33358452 10.1016/j.psym.2020.05.014

[63] Goldstein LH, Drew C, Mellers J, Mitchell-O'Malley S, Oakley DA. Dissociation, hypnotizability, coping styles and health locus of control: characteristics of pseudoseizure patients. Seizure 2000; 9(5): 314–22. 10933985 10.1053/seiz.2000.0421

[64] Goldstein LH, Mellers JD. Ictal symptoms of anxiety, avoidance behaviour, and dissociation in patients with dissociative seizures. J Neurol Neurosurg Psychiatry 2006; 77: 616–21. 16614021 10.1136/jnnp.2005.066878 PMC2117432

[65] González-Vázquez AI, Del Río-Casanova L, Seijo-Ameneiros N, Cabaleiro-Fernández P, Seoane-Pillado T, Justo-Alonso A, Validity and reliability of the Spanish version of the Somatoform Dissociation Questionnaire (SDQ-20). Psicothema 2017; 29: 275–80. 28438254 10.7334/psicothema2016.346

[66] Guz H, Doganay Z, Ozkan A, Colak E, Tomac A, Sarisoy G. Conversion disorder and its subtypes: a need for a reclassification. Nord J Psychiatry 2003; 57: 377–81. 14522603 10.1080/08039480310002723

[67] Guz H, Doganay Z, Ozkan A, Colak E, Tomac A, Sarisoy G. Conversion and somatization disorders; dissociative symptoms and other characteristics. J Psychosom Res 2004; 56: 287–91. 15046964 10.1016/S0022-3999(03)00069-2

[68] Güleç MY, Ýnanç L, Yanartaþ Ö, Üzer A, Güleç H. Predictors of suicide in patients with conversion disorder. Compr Psychiatry 2014; 55: 457–62. 24269192 10.1016/j.comppsych.2013.10.009

[69] Hammond-Tooke GD, Grajeda FT, Macrorie H, Franz EA. Response inhibition in patients with functional neurological symptom disorder. J Clin Neurosci 2018; 56: 38–43. 30145086 10.1016/j.jocn.2018.08.005

[70] Herrero H, Tarrada A, Haffen E, Mignot T, Sense C, Schwan R, Skin conductance response and emotional response in women with psychogenic non-epileptic seizures. Seizure 2020; 81: 123–31. 32795943 10.1016/j.seizure.2020.07.028

[71] Holper S, Foster E, Lloyd M, Rayner G, Rychkova M, Ali R, Clinical predictors of discordance between screening tests and psychiatric assessment for depressive and anxiety disorders among patients being evaluated for seizure disorders. Epilepsia 2021; 62: 1170–83. 33735445 10.1111/epi.16871

[72] Irorutola F, Gerhardt C, Hamouda K, Rose M, Hinkelmann K, Senf-Beckenbach P. Emotional and cognitive empathy in patients with non-epileptic seizures. Seizure 2020; 81: 280–6. 32927242 10.1016/j.seizure.2020.08.009

[73] Jalilianhasanpour R, Ospina JP, Williams B, Mello J, MacLean J, Ranford J, Secure attachment and depression predict 6-month outcome in motor functional neurological disorders: a prospective pilot study. Psychosomatics 2019; 60: 365–75. 30342702 10.1016/j.psym.2018.08.004

[74] Jungilligens J, Ospina JP, Williams B, Mello J, MacLean J, Ranford J, Impaired emotional and behavioural awareness and control in patients with dissociative seizures. Psychol Med 2020; 50: 2731–9. 31625504 10.1017/S0033291719002861

[75] Kienle J, Rockstroh B, Bohus M, Fiess J, Huffziger S, Steffen-Klatt A. Somatoform dissociation and posttraumatic stress syndrome - two sides of the same medal? A comparison of symptom profiles, trauma history and altered affect regulation between patients with functional neurological symptoms and patients with PTSD. BMC Psychiatry 2017; 17(1): 248. 28693577 10.1186/s12888-017-1414-z PMC5504809

[76] Koreki A, Garfkinel SN, Mula M, Agrawal N, Cope S, Eilon T, Trait and state interoceptive abnormalities are associated with dissociation and seizure frequency in patients with functional seizures. Epilepsia 2020; 61: 1156–65. 32501547 10.1111/epi.16532 PMC7737228

[77] Kranick S, Ekanayake V, Martinez V, Ameli R, Hallett M, Voon V. Psychopathology and psychogenic movement disorders. Mov Disord 2011; 26: 1844–50. 21714007 10.1002/mds.23830 PMC4049464

[78] Kuyk J, Siffels MC, Bakvis P, Swinkels WA. Psychological treatment of patients with psychogenic non-epileptic seizures: an outcome study. Seizure 2008; 17: 595–603. 18395473 10.1016/j.seizure.2008.02.006

[79] Martino I, Psychopathological constellation in patients with PNES: a new hypothesis. Epilepsy Behav 2018; 78: 297–301. 29092782 10.1016/j.yebeh.2017.09.025

[80] Martino I, Bruni A, Labate A, Vasta R, Cerasa A, Borzì G, The impact of sexual abuse on psychopathology of patients with psychogenic nonepileptic seizures. Neurol Sci 2021; 42: 1423–8. 32794127 10.1007/s10072-020-04652-7

[81] Mitchell JW, Ali F, Cavanna AE. Dissociative experiences and quality of life in patients with non-epileptic attack disorder. Epilepsy Behav 2012; 25: 307–12. 23099232 10.1016/j.yebeh.2012.08.022

[82] Mousa S, Latchford G, Weighall A, Nash H, Murray-Leslie R, Reuber M, Evidence of objective sleep impairment in nonepileptic attack disorder: a naturalistic prospective controlled study using actigraphy and daily sleep diaries over six nights. Epilepsy Behav 2021; 117: 107867. 33684785 10.1016/j.yebeh.2021.107867

[83] Myers L, Trobliger R, Bortnik K, Zeng R, Saal E, Lancman M. Psychological trauma, somatization, dissociation, and psychiatric comorbidities in patients with psychogenic nonepileptic seizures compared with those in patients with intractable partial epilepsy. Epilepsy Behav 2019; 92: 108–13. 30654229 10.1016/j.yebeh.2018.12.027

[84] Nisticò V, Caputo G, Tedesco R, Marzorati A, Ferrucci R, Priori A, Dissociation during Mirror Gazing Test in psychogenic nonepileptic seizures and functional movement disorders. Epilepsy Behav 2020; 112: 107368. 32861024 10.1016/j.yebeh.2020.107368

[85] Ozcetin A, Belli H, Ertem U, Bahcebasi T, Ataoglu A, Canan F. Childhood trauma and dissociation in women with pseudoseizure-type conversion disorder. Nord J Psychiatry 2009; 63: 462–8. 19544219 10.3109/08039480903029728

[86] Ozdemir PG, Gur T, Cokluk E, Isik M, Tapan S. Vitamin B12, folate levels and somatoform dissociation in conversion disorder. J Pak Med Assoc 2020; 70: 1758–61. 33159748 10.5455/JPMA.31213

[87] O'Brien FM, Fortune GM, Dicker P, O'Hanlon E, Cassidy E, Delanty N, Psychiatric and neuropsychological profiles of people with psychogenic nonepileptic seizures. Epilepsy Behav 2015; 43: 39–45. 25553390 10.1016/j.yebeh.2014.11.012

[88] Perez DL, Matin N, Williams B, Tanev K, Makris N, LaFrance WC Jr, Cortical thickness alterations linked to somatoform and psychological dissociation in functional neurological disorders. Hum Brain Mapp 2018; 39: 428–39. 29080235 10.1002/hbm.23853 PMC5747307

[89] Pick S, Rojas-Aguiluz M, Butler M, Mulrenan H, Nicholson TR, Goldstein LH. Dissociation and interoception in functional neurological disorder. Cogn Neuropsychiatry 2020; 25: 294–311. 32635804 10.1080/13546805.2020.1791061

[90] Pick S, Mellers JD, Goldstein LH. Dissociation in patients with dissociative seizures: relationships with trauma and seizure symptoms. Psychol Med 2017; 47: 1215–29. 28065191 10.1017/S0033291716003093

[91] Proença IC, Castro LH, Jorge CL, Marchetti RL. Emotional trauma and abuse in patients with psychogenic nonepileptic seizures. Epilepsy Behav 2011; 20: 331–3. 21315658 10.1016/j.yebeh.2010.11.015

[92] Reedijk WB, van Rijn MA, Roelofs K, Tuijl JP, Marinus J, van Hilten JJ. Psychological features of patients with complex regional pain syndrome type I related dystonia. Mov Disord 2008; 23: 1551–9. 18546322 10.1002/mds.22159

[93] Reuber M, House AO, Pukrop R, Bauer J, Elger CE. Somatization, dissociation and general psychopathology in patients with psychogenic non-epileptic seizures. Epilepsy Res 2003; 57: 159–67. 15013057 10.1016/j.eplepsyres.2003.11.004

[94] Reuber M, Pukrop R, Mitchell AJ, Bauer J, Elger CE. Clinical significance of recurrent psychogenic nonepileptic seizure status. J Neurol 2003; 250: 1355–62. 14648153 10.1007/s00415-003-0224-z

[95] Roelofs K, Keijsers GP, Hoogduin KA, Näring GW, Moene FC. Childhood abuse in patients with conversion disorder. Am J Psychiatry 2002; 159: 1908–13. 12411227 10.1176/appi.ajp.159.11.1908

[96] Spinhoven P, Roelofs K, Moene F, Kuyk J, Nijenhuis E, Hoogduin K, Trauma and dissociation in conversion disorder and chronic pelvic pain. Int J Psychiatry Med 2004; 34: 305–18. 15825581 10.2190/YDK2-C66W-CL6L-N5TK

[97] Spitzer C, Spelsberg B, Grabe HJ, Mundt B, Freyberger HJ. Dissociative experiences and psychopathology in conversion disorders. J Psychosom Res 1999; 46: 291–4. 10193920 10.1016/s0022-3999(98)00112-3

[98] Steffen A, Fiess J, Schmidt R, Rockstroh B. “That pulled the rug out from under my feet!” – adverse experiences and altered emotion processing in patients with functional neurological symptoms compared to healthy comparison subjects. BMC Psychiatry 2015; 15: 133. 26103961 10.1186/s12888-015-0514-x PMC4477601

[99] Steffen-Klatt A, Fiess J, Beckha J, Schmidt R, Rockstroh B. The impact of adverse childhood experience on symptom severity in patients with functional neurological symptom disorder (FNSD). Ment Health Prev 2019; 13: 169–75.

[100] Stins JF, Kempe CL, Hagenaars MA, Beek PJ, Roelofs K. Attention and postural control in patients with conversion paresis. J Psychosom Res 2015; 78: 249–54. 25466324 10.1016/j.jpsychores.2014.11.009

[101] van der Hoeven RM, Broersma M, Pijnenborg GH, Koops EA, van Laar T, Stone J, Functional (psychogenic) movement disorders associated with normal scores in psychological questionnaires: a case control study. J Psychosom Res 2015; 79: 190–4. 26113484 10.1016/j.jpsychores.2015.06.002

[102] van der Kruijs SJ, Jagannathan SR, Bodde NM, Besseling RM, Lazeron RH, Vonck KE, Resting-state networks and dissociation in psychogenic non-epileptic seizures. J Psychiatr Res 2014; 54: 126–33. 24703187 10.1016/j.jpsychires.2014.03.010

[103] Walther K, Volbers B, Erdmann L, Dogan Onugoren M, Gollwitzer S, Kasper BS, Psychological long-term outcome in patients with psychogenic nonepileptic seizures. Epilepsia 2019; 60: 669–78. 30838655 10.1111/epi.14682

[104] Williams B, Ospina JP, Jalilianhasanpour R, Fricchione GL, Perez DL. Fearful attachment linked to childhood abuse, alexithymia, and depression in motor functional neurological disorders. J Neuropsychiatry Clin Neurosci 2019; 31: 65–9. 30376786 10.1176/appi.neuropsych.18040095 PMC6349486

[105] Wood BL, McDaniel S, Burchfiel K, Erba G. Factors distinguishing families of patients with psychogenic seizures from families of patients with epilepsy. Epilepsia 1998; 39: 432–7. 9578034 10.1111/j.1528-1157.1998.tb01396.x

[106] Xue Q, Wang ZY, Xiong XC, Tian CY, Wang YP, Xu P. Altered brain connectivity in patients with psychogenic non-epileptic seizures: a scalp electroencephalography study. J Int Med Res 2013; 41: 1682–90. 24026773 10.1177/0300060513496170

[107] Carlson E, Putnam FW, Ross CA, Torem M, Coons P, Dill DL, Validity of the Dissociative Experiences Scale in screening for multiple personality disorder: a multicenter study. Am J Psychiatry 1993; 150: 1030–6. 8317572 10.1176/ajp.150.7.1030

[108] Briere J. Trauma Symptom Inventory-2: Professional Manual. PAR, 2011.

[109] Sierra M, Berrios GE. The Cambridge Depersonalisation Scale: a new instrument for the measurement of depersonalisation. Psychiatry Res 2000; 93: 153–64. 10725532 10.1016/s0165-1781(00)00100-1

[110] Sanders S. The perceptual alteration scale: a scale measuring dissociation. Am J Clin Hypn 1986; 29: 95–102. 3776897 10.1080/00029157.1986.10402691

[111] Kennedy F, Clarke S, Stopa L, Bell L, Rouse H, Ainsworth C, Towards a cognitive model and measure of dissociation. J Behav Ther Exp Psychiatry 2004; 35: 25–48. 15157816 10.1016/j.jbtep.2004.01.002

[112] Freyberger HJ, Spitzer C, Stieglitz RD, Kuhn G, Magdeburg N, Bernstein-Carlson E. Fragebogen zu dissoziativen Symptomen (FDS). deutsche adaptation, reliabilität und validität der Amerikanischen Dissociative Experience Scale (DES) [Questionnaire on dissociative symptoms. German adaptation, reliability and validity of the American Dissociative Experience Scale (DES)]. Psychother Psychosom Med Psychol 1998; 48: 223–9. 9677826

[113] Nijenhuis ER. The scoring and interpretation of the SDQ-20 and SDQ-5. Act Nerv Super (Praha) 2010; 52: 24–8.

[114] Gratz KL, Roemer L. Multidimensional assessment of emotion regulation and dysregulation: development, factor structure, and initial validation of the difficulties in emotion regulation scale. J Psychopathol Behav Assess 2004; 26: 41–54.

[115] Gagny M, Grenevald L, El-Hage W, Chrusciel J, Sanchez S, Schwan R, Explanatory factors of quality of life in psychogenic non-epileptic seizure. Seizure 2021; 84: 6–13. 33254100 10.1016/j.seizure.2020.10.028

[116] Devinsky O, Vickrey BG, Cramer J, Perrine K, Hermann B, Meador K, Development of the Quality of Life in Epilepsy Inventory. Epilepsia 1995; 36: 1089–104. 7588453 10.1111/j.1528-1157.1995.tb00467.x

[117] Kienle J, Rockstroh B, Fiess J, Schmidt R, Popov T, Steffen-Klatt A. Variation of functional neurological symptoms and emotion regulation with time. Front Psychiatry 2018; 9: 35. 29487543 10.3389/fpsyt.2018.00035 PMC5816796

[118] van der Kruijs SJ, Bodde NM, Vaessen MJ, Lazeron RH, Vonck K, Boon P, Functional connectivity of dissociation in patients with psychogenic non-epileptic seizures. J Neurol Neurosurg Psychiatry 2012; 83: 239–47. 22056967 10.1136/jnnp-2011-300776

[119] Boucsein W. Electrodermal Activity. Springer Science & Business Media, 2012.

[120] Krüger C, Mace CJ. Psychometric validation of the state scale of dissociation (SSD). Psychol Psychother 2002; 75: 33–51. 12006198 10.1348/147608302169535

[121] Şar V, Akyüz G, Doğan O. Prevalence of dissociative disorders among women in the general population. Psychiatry Res 2007; 149: 169–76. 17157389 10.1016/j.psychres.2006.01.005

[122] Spitzer C, Liss H, Dudeck M, Orlob S, Gillner M, Hamm A, Dissociative experiences and disorders in forensic inpatients. Int J Law Psychiatry 2003; 26: 281–8. 12689627 10.1016/S0160-2527(03)00038-4

[123] Sar V. Epidemiology of dissociative disorders: an overview. Epidemiol Res Int 2011; 404538.

[124] Kate M-A, Hopwood T, Jamieson G. The prevalence of dissociative disorders and dissociative experiences in college populations: a meta-analysis of 98 studies. J Trauma Dissociation 2020; 21: 16–61. 31461395 10.1080/15299732.2019.1647915

[125] Foote B, Smolin Y, Kaplan M, Legatt ME, Lipschitz D. Prevalence of dissociative disorders in psychiatric outpatients. Am J Psychiatry 2006; 163: 623–9. 16585436 10.1176/ajp.2006.163.4.623

[126] Ross CA, Duffy CM, Ellason JW. Prevalence, reliability and validity of dissociative disorders in an inpatient setting. J Trauma Dissociation 2002; 3: 7–17.

[127] Tutkun H, Sar V, Yargiç LI, Ozpulat T, Yanik M, Kiziltan E. Frequency of dissociative disorders among psychiatric inpatients in a Turkish university clinic. Am J Psychiatry 1998; 155: 800–5. 9619153 10.1176/ajp.155.6.800

[128] Cope SR. EMDR as an adjunctive psychological therapy for patients with functional neurological disorder: illustrative case examples. J EMDR Prac Res 2020; 2: 76–89.

[129] Fine CG, Berkowitz AS. The wreathing protocol: the imbrication of hypnosis and EMDR in the treatment of dissociative identity disorder and other dissociative responses. Am J Clin Hypn 2001; 43: 275–90. 11269630 10.1080/00029157.2001.10404282

[130] International Society for the Study of Trauma and Dissociation. Guidelines for Treating Dissociative Identity Disorder in Adults, Third Revision. J Trauma Dissociation 2011; 12: 115–87. 21391103 10.1080/15299732.2011.537247

[131] Cope SR, Mountford L, Smith JG, Agrawal N EMDR to treat functional neurological disorder: a review. J EMDR Prac Res 2018; 3: 118–32.

[132] Malda Castillo J, Beton E, Coman C, Howell B, Burness C, Martlew J, Three sessions of intensive short-term dynamic psychotherapy (ISTDP) for patients with dissociative seizures: a pilot study. Psychoanal Psychother 2022; 36: 81–104.

[133] Russell LA, Abbass AA, Allder SJ, Kisely S, Pohlmann-Eden B, Town JM. A pilot study of reduction in healthcare costs following the application of intensive short-term dynamic psychotherapy for psychogenic nonepileptic seizures. Epilepsy Behav 2016; 63: 17–9. 27541836 10.1016/j.yebeh.2016.07.017

[134] Nijenhuis ER. Somatoform dissociation: major symptoms of dissociative disorders. J Trauma Dissociation 2001; 1(4): 7–32.

[135] Van IJzendoorn MH, Schuengel C. The measurement of dissociation in normal and clinical populations: meta-analytic validation of the Dissociative Experiences Scale (DES). Clin Psychol Rev 1996; 16: 365–82.

[136] Lawton G, Baker GA, Brown RJ. Comparison of two types of dissociation in epileptic and nonepileptic seizures. Epilepsy Behav 2008; 13: 333–6. 18514035 10.1016/j.yebeh.2008.04.015

[137] Briere J, Weathers FW, Runtz M. Is dissociation a multidimensional construct? Data from the multiscale dissociation inventory. J Trauma Stress 2005; 18: 221–31. 16281216 10.1002/jts.20024

[138] Drane DL, Fani N, Hallett M, Khalsa SS, Perez DL, Roberts NA. A framework for understanding the pathophysiology of functional neurological disorder. CNS Spectr [Epub ahead of print] 4 Sep 2021. Available from: 10.1017/S1092852920001789. PMC7930164 32883381

[139] Lanius RA, Vermetten E, Loewenstein RJ, Brand B, Schmahl C, Bremner JD, Emotion modulation in PTSD: clinical and neurobiological evidence for a dissociative subtype. Am J Psychiatry 2010; 167: 640–7. 20360318 10.1176/appi.ajp.2009.09081168 PMC3226703

[140] Reinders AATS, Willemsen AT, den Boer JA, Vos HP, Veltman DJ, Loewenstein RJ. Opposite brain emotion-regulation patterns in identity states of dissociative identity disorder: a PET study and neurobiological model. Psychiatry Res 2014; 223: 236–43. 24976633 10.1016/j.pscychresns.2014.05.005

[141] Osimo EF, Pillinger T, Rodriguez IM, Khandaker GM, Pariante CM, Howes OD. Inflammatory markers in depression: a meta-analysis of mean differences and variability in 5,166 patients and 5,083 controls. Brain, Behavior, and Immunity 2020; 87: 901–9. 32113908 10.1016/j.bbi.2020.02.010 PMC7327519

[142] Perez DL, Nicholson TR, Asadi-Pooya AA, Bègue I, Butler M, Carson AJ, Neuroimaging in functional neurological disorder: state of the field and research agenda. Neuroimage 2021; 30: 102623. 34215138 10.1016/j.nicl.2021.102623 PMC8111317

[143] Ospina JP, Jalilianhasanpour R, Perez DL. The role of the anterior and midcingulate cortex in the neurobiology of functional neurologic disorder. Handb Clin Neurol 2019; 166: 267–79. 31731915 10.1016/B978-0-444-64196-0.00014-5 PMC7012371

[144] Wegrzyk J, Kebets V, Richiardi J, Galli S, de Ville DV, Aybek S. Identifying motor functional neurological disorder using resting-state functional connectivity. Neuroimage 2018; 17: 163–8. 29071210 10.1016/j.nicl.2017.10.012 PMC5651543

[145] Faul L, Knight LK, Espay AJ, Depue BE, LaFaver K. Neural activity in functional movement disorders after inpatient rehabilitation. Psychiatry Res 2020; 303: 111125. 10.1016/j.pscychresns.2020.111125 32585576

[146] Espay AJ, Ries S, Maloney T, Vannest J, Neefus E, Dwivedi AK, Clinical and neural responses to cognitive behavioral therapy for functional tremor. Neurology 2019; 93: e1787–98. 31586023 10.1212/WNL.0000000000008442 PMC6946484

[147] Labate A, Cerasa A, Mula M, Mumoli L, Gioia MC, Aguglia U, Neuroanatomic correlates of psychogenic nonepileptic seizures: a cortical thickness and VBM study. Epilepsia 2012; 53: 377–85. 22150750 10.1111/j.1528-1167.2011.03347.x

[148] Labate A, Martino I, Caligiuri ME, Fortunato F, Bruni A, Segura-Garcia C, Orbito-frontal thinning together with a somatoform dissociation might be the fingerprint of PNES. Epilepsy Behav 2021; 121(Pt A): 108044. 34051606 10.1016/j.yebeh.2021.108044

[149] Kılıç F, Coşkun M, Bozkurt H, Kaya İ, Zoroğlu S. Self-injury and suicide attempt in relation with trauma and dissociation among adolescents with dissociative and non-dissociative disorders. Psychiatry Invest 2017; 14: 172–8. 10.4306/pi.2017.14.2.172 PMC5355015 28326115

