# Supplementary material for: Dissociation and its biological and clinical associations in functional neurological disorder: systematic review and meta-analysis
Source: BJPsych Open. 2022 Dec 1;9(1):e2. doi: 10.1192/bjo.2022.597 (PMC9798224; doi:10.1192/bjo.2022.597)

Contents of Supplementary File

Supplementary

[Supplementary Box 1: Example search strategy including Boolean operators 2](#_Toc102644414)

[Supplementary Table 1: ICD and DSM codes for FND and Dissociative Disorders 3](#_Toc102644415)

[Supplementary Table 2: Summary of Newcastle Ottawa Total Scores of Included Studies 4](#_Toc102644416)

[Supplementary Table 3: Additional Description and Overview of Eligible Studies 5](#_Toc102644417)

[Supplementary Table 4: Newcastle Ottawa Scale Ratings for Case-control Studies 45](#_Toc102644418)

[Supplementary Table 5: Adapted Newcastle Ottawa Scale Ratings for Cross-sectional Studies 52](#_Toc102644419)

[Supplementary Table 6: Newcastle Ottawa Scale Ratings for Cohort Studies 56](#_Toc102644420)

[Supplementary Figure 1: Funnel plot of SDQ-20 scores 58](#_Toc102644421)

[Supplementary Figure 2: Forest plot of SDQ-20 scores with Demartini et al. removed 59](#_Toc102644422)

[Supplementary Figure 3: Funnel plot of SDQ-20 scores with Demartini et al. removed 60](#_Toc102644423)

[Supplementary Figure 4: Funnel plot of Psychoform Dissociation Studies 61](#_Toc102644424)

[Supplementary Figure 5: Funnel plot for Psychoform Dissociation in FND seizure Vs FND motor subgroups 62](#_Toc102644425)

### Supplementary Box 1: Example search strategy including Boolean operators

| - 1. ”functional neurological"[Title/Abstract] OR   2. "functional motor"[Title/Abstract] OR   3. "conversion disorder"[Title/Abstract] OR   4. "psychogenic seizure"[Title/Abstract] OR   5. "pseudoseizure"[Title/Abstract] OR   6. "dissociative seizure"[Title/Abstract])) AND   7. ("dissociative disorder"[Title/Abstract] OR   8. dissociative[Title/Abstract] OR   9. depersonalization[Title/Abstract] OR   10. depersonalisation[Title/Abstract] OR 11.   11. derealisation[Title/Abstract] OR 12.   12. derealization[Title/Abstract])) AND 13.   13. "last 40 years"[PDat]) |
| --- |

### Supplementary Table 1: ICD and DSM codes for FND and Dissociative Disorders

| **Dissociative [conversion] Disorders** | **ICD Code** | **DSM Code** |
| --- | --- | --- |
| Dissociative amnesia | F44.0 | 300.12 |
| Dissociative fugue | F44.1 | 300.13 |
| Dissociative stupor | F44.2 | N/A |
| Trance and possession disorders | F44.3 | N/A |
| Dissociative motor disorders | F44.4 | 300.11 |
| Dissociative convulsions | F44.5 | 300.11 |
| Dissociative anaesthesia and sensory loss | F44.6 | 300.11 |
| Mixed dissociative [conversion] disorders | F44.7 | 300.11 |
| Other dissociative [conversion] disorders | F44.8 | 300.15 |
| Dissociative [conversion] disorder, unspecified | F44.9 | 300.15 |
| **Other Neurotic Disorders** | **ICD Code** | **DSM Code** |
| Depersonalization-Derealization Syndrome | F48.1 | 300.6 |

### Supplementary Table 2: Summary of Newcastle Ottawa Total Scores of Included Studies

| Included study’s risk of bias according to Newcastle Ottawa Scale Total Score Categories | | | |
| --- | --- | --- | --- |
|  | Very High | High | Low |
| Case Control | 2 | 32 | 20 |
| Cohort | 1 | 5 | - |
| Cross-sectional | 5 | 10 | - |
| Please note the Newcastle Ottawa scale for case-control and cohort studies has a range of 0-9, while cross-sectional is 0-8.  Wells, G.A., et al., *The Newcastle-Ottawa Scale (NOS) for assessing the quality of nonrandomised studies in meta-analyses*. 2000, Oxford. A study with score from 7-9 has high quality, 4-6 high risk, and 0-3 very high risk of bias. | | | |

### Supplementary Table 3: Additional Description and Overview of Eligible Studies

| Overview of Eligible Studies | | | | | |
| --- | --- | --- | --- | --- | --- |
| **Author** | **Participant characteristics** | **Design / Aims / Outcomes** | **Dissociation scales** | **Key findings** | **Strengths & Limitations** |
| Gagny et al., 2021 | FND, FND-Seizures (n = 107);  female n = 81; male n = 51  Gender ratio 3.12  Mean age 33.7 | Cohort  Quality of life in Non-epileptic Seizures  QOLIE-31 sub-scale | DES | No statistical influence of DES on QoL scores | No control comparison  Does not present raw data for scores  Diagnosis EEG-confirmed  Possible confounding effect of medications not assessed |
| Gerhardt et al., 2021 | FND, FND-Seizures (n = 40);  HC (n = 44) | Case-control  Attachment in adult patients with PNES with a focus on the role of unresolved/disorganized attachment | SDQ | No difference in dissociation symptoms between FND-Seizures patients with organized or disorganised attachment | Diagnosis EEG confirmed  Possible confounding effect of medications  Did not control for confounders such as age or gender |
| Holper et al., 2021 | FND, FND-Seizures (n = 62)  Epilepsy (n= 234) | Cross-sectional  Investigated factors that predict discordance between screening instruments (NDDI- E and GAD- 7), and diagnoses made by qualified psychiatrists among patients with seizure disorders | Wessex Dissociation Scale | Seven clinical factors were predictive of discordant screening for both depression and anxiety: including greater dissociative symptoms, greater patient-reported adverse events, subjective cognitive impairment, negative affect, detachment, disinhibition, and psychoticism | Patient sample derived from those admitted for inpatient VEED  Did not control for confounders such as age or medication |
| Cope et al., 2017 | FND-seizures (n = 16) (subset of wider group with comorbid epilepsy; data requested from author) | Pilot study: CBT-based psychoeducation for individuals with FND-seizures. Baseline DES was measured in subjects. | DES | Patient understanding, functional wellbeing, and attack-frequency decreased following the CBT intervention | Did not separate FND-seizures subjects with comorbid epilepsy |
| Mousa et al., 2021 | FND FND-Seizures (n= 17)  HC (n= 20) | Case-control  Study designed to investigate the extent of subjective and objective sleep impairments in those with FND-Seizures  Actigraphy and sleep diary | DES | Explored whether reported sleep disturbance in NEAD is better considered objective or subjective and not the possible reasons for any observed sleep impairment  Highlighted the difficulty finding a valid and practical daily measure of dissociation  The FND-Seizures group reported higher levels of dissociation than those in the control group | Diagnosis given by trained neurologist at specialist epilepsy service and EEG if available  Excluded patients with mixed seizure disorder or previous diagnosis of a sleep disorder  Controls were recruited from a university volunteer mailing list  Control group was matched by gender and age (but not employment)  Non-respondent data is described  Small sample makes generalisation difficult |
| Herrero et al., 2020 | FND-Seizures (n = 34)  HC (n = 34) | Case-control  Skin conductance response (SCR) (rate, amplitude and latency), heart rate deceleration and emotional response in women with psychogenic non-epileptic seizures | DES | FND-Seizures physiological response (SCR and heart rate deceleration) was negatively correlated to dissociation tendency (r=-0.48, p = 0.0083)  Results suggest that dissociation and difficulty in describing feelings are associated with an altered physiological response in PNES women only | Female population only  Controls matched for age and level of education  Diagnosis confirmed by EEG  Data on medication was collected (9% normal volunteers were under psychotropic treatment compared to 52.9% in the PNES group but this was not controlled when assessing dissociation  Small sample make generalisation difficult |
| Koreki et al.,  2020 | FND, FND-Seizures (41)  HC (30)  Mean age 32  F:M ratio 19.5:1 Outpatient setting | Case / control study  FND-Seizures vs healthy controls  Dissociation, seizure frequency and interoceptive abnormalities  Heartbeat detection task | SDQ-20  MDI- DP | Mean SDQ-20: 38 ± 12.8; significantly greater than control (p=0.001).  Psychoform and somatoform dissociation negatively associated with interoceptive ability; this in turn was associated with frequency of seizures | Measures of somatoform and psychoform dissociation used  Not video-EEG confirmed  FND sample not consecutively recruited  Did not exclude active severe psychiatric comorbidity |
| Irorutola et al., 2020 | FND FND-Seizures (n= 41)  HC (n= 41) | Case-control  Investigated impairments in social cognition in the form of emotional and cognitive empathy in patients with NES compared to healthy controls | Full version FDS | FND-Seizures patients showed higher dissociation symptoms than the healthy controls  Symptom severity of dissociative disorders measured with the FDS is a significant predictor for impairments of emotional empathy regarding positive emotions (p < 0.01) | Small sample makes generalisation difficult  VEEG confirmed diagnosis  Controls matched by age, gender and education level  Patients with comorbid epilepsy excluded  Dissociation measure incomparable to DES (extended version of DES) |
| Jungilligens et al., 2020 | FND FND-Seizures (n=20)  HC (n= 20) | Case-control  Assessed specific metacognitive traits and behavioural features involved in the affective and cognitive underpinnings of patients with FND-Seizures (emotion recognition and regulation, inhibition, interoception and sense of agency) | FDS  (Comparable to DES)  SDQ | Symptoms of somatoform dissociation as measured with the SDQ were significantly higher in FND-Seizures group (p< 0.001)  No significant group differences concerning the DES (p= 0.18)  DES and SDQ measures did not correlate with experimental measures of behavioural or bodily awareness | Inpatient only  VEEG confirmed FND-Seizures  No-significant differences in age or gender between groups  Small sample size makes generalisation difficult  Possible confounding effect of medications not assessed |
| Martino et al., 2020 | FND FND-Seizures (n = 63) | Cross-sectional  The impact of sexual abuse on psychopathology of patients with psychogenic nonepileptic seizures  Compared those with and without sexual assault history | DES | Patients with history of sexual abuse showed higher dissociation scores on the DES (p= 0.003)  No difference in somatoform dissociation scores between those with and without a history of sexual assault (p=0.49)  An interaction effect suggested patients with a history of sexual assault and daily seizures tended to show higher dissociation scores (DES) (p= 0.012) | With and without sexual assault groups  VEEG confirmed diagnosis  Excluded patient with comorbid epilepsy  Possible confounding effect of medications not assessed |
| Nistico et al., 2020 | FND FND-Seizures (n= 11)  FND Motor (n= 17)  HC (n= 18) | Case-control  Comparison between FND symptom subtypes  Assessed dissociation via the Mirror Gazing Test and the Strange Face Questionnaire (ad-hoc questionnaire on sensations and perceptions participants experienced in the Mirror) | CADSS | FND-Seizures, FMD, and HCs did total scores did not differ on the Strange Face Questionnaire (p = 0.011)  FND-Seizures scored higher than HCs at the Strange Face Questionnaire on subscale Dissociative Identity/Compartmentalization (p =0.03), CADSS Total Score and its subscale Dissociative Amnesia (p = 0.025)  FMD patient scored higher the HCs on the CADSS Depersonalisation subscale (p = 0.043) | Small sample makes generalisation difficult  Did not control for medication  Dependent upon the validity of the Mirror Gazing Test  FND-Seizures diagnosis confirmed by VEEG  FMD diagnosis given by specialist  Excluded patients with comorbid epilepsy or overlay between functional and organic movement disorders |
| Ozdemir et al., 2020 | FND mixed (n = 55)  HC (n= 45) | Case-control  Folate and vitamin B12 levels in patients with FND  Depression and dissociation examined | SDQ | B12 in FND differed (M = 283.93, SD ±122.96) compared to HC (M = 324.62, SD ± 128.82, p=0.05)  The mean level of folic acid in FND patients was 5.47 (SD ± 1.84) and 6.07 (SD ± 2.26) in HCs, this did not statistically differ  SDQ scores were higher in the FND group compared to controls (p=.001) | Sample age restricted to under 50 years  Limited description of diagnostic procedure except according to DSM-5 and a “normal neurological examination”  Did not control for medication  No controlling of age or gender |
| Pick et al., 2020 | FND mixed (n = 19)  HC (n= 20) | Case-control  Susceptibility to dissociation and the impact of dissociation on interoceptive processing in individuals with FND  Heartbeat-tracking task measuring interoception  Exteroceptive processing control task | CADSS | FND patients had higher levels of dissociation at baseline compared to HCs (p = 0.001)  Dissociation levels increased following dissociation-induction task  Interoceptive accuracy did not differ between groups at baseline, but the FND group had lower accuracy post dissociation induction (p= 0.021)  Confidence ratings on interoceptive and exteroceptive processing tasks were lower in the FND group (p < 0.05) | First examination of the influence of dissociative states on interoception in FND  Small sample makes generalisation difficult  Dissociation induction dependent upon Mirror Gazing Task  Diagnosis given by specialist  Recorded but did not control for impact of medication  Did not control for factors such as age or gender |
| Sarudiansky et al, 2020 | FND, FND-Seizures (n = 12) | Cross-sectional  Psycho-educational intervention in patients with FND-Seizures | DES | No significant reduction in dissociation symptoms following psychoeducational intervention | VEEG confirmed diagnosis  Small sample makes generalisation difficult  Non-respondent data described  No control group  Did not control for age or gender  Non-consecutive sampling |
| Walther et al., 2019 | FND, FND-Seizures (n = 52);  FND-Seizures symptoms resolved (n=19); gender ratio F:M 2.16:1; average age  33.6  FND-Seizures symptoms ongoing (n=33); gender ratio F:M 3.71:1; average age 44.4  Outpatient setting | Cohort study  Long-term outcome in FND-Seizures patients  Impact of dissociation on outcome  psychopathology | DES | Median DES score higher in patients with ongoing FND-Seizures (n = 33) compared to patients who have remitted (n = 19) (p = 0.05) | Follow up over 3 to 5 years  Moderate sample size  Considerable loss of participants to follow up  FND sample not consecutively recruited  Possible confounding effect of medications not assessed |
| Jalilianhasanpour et al., 2019 | FND; mixed (n = 34)  Mean age 41.3; gender ratio F:M 28:6  Outpatient setting | Cohort prospective study – baseline and 6-month assessment were conducted of a mixed FND cohort following education and introduction of CBT indi-  vidualized treatment plan emphasizing  CBT, PT, and OT. | DES | DES was not examined relative to 6-month outcomes.  Baseline secure attachment traits and depression as  measured by the Relationship Scales Questionnaire and Beck  Depression Inventory-II positively correlated with improved  Patient Health Questionnaire-15 scores. | Examined a wide array of neuropsychiatric characteristics  Did not assess re-record DES score at follow-up |
| Myers et al.,  2019 | FND, FND-Seizures (n = 161)  Intractable ES (n = 96)  FND-Seizures F:M gender ratio 1.98:1  Mean age 35 Mixed setting | Case/Control  ES vs FND-Seizures psychological trauma, somatization, dissociation, and comorbidities  Trauma history  Dissociation score | TSI | Childhood sexual abuse was significantly higher in FND-Seizures compared to ES (30.4% vs 11.5%; p = 0.002)  FND-Seizures patients did not differ on TSI- Dissociation from ES | Did not exclude severe psychiatric comorbidity  Possible confounding effect of medications not assessed  Possible confounding effect of medications not assessed |
| Williams et al., 2019 | FND; mixed sample (n=54)  FND F:M Gender ratio 2.73:1  Mean age 40.2  Outpatient setting | FND only cohort study  Insecure attachment in FND patients  Dissociation scores | DES  SDQ-20 | Mean DES = 19.2 ± 14.4  Mean SDQ-20 = 32.2  ± 10.10  DES and SDQ positively correlated with fearful attachment (r=0.57, p<0.001; r=0.43 p=0.001, respectively) | Measured psychoform and somatoform dissociation  Lack of control group  FND diagnosis not explicitly made by specialist  Possible confounding effect of medications not assessed  FND sample not consecutively recruited  Not video-EEG confirmed |
| Steffen-Klatt  et al., 2019 | FND, multiple (82)  HC (82)  Mean Age 41.63  F:M 2.72:1  Outpatient setting | Case / control  Adverse childhood experiences potential impact on depression, alexithymia and functional symptoms  FND severity evaluated by SDQ-20 score | SDQ-20 | Median SDQ-20: 30 (IQR 9)  More abuse and neglect reported in FND group  Multiple linear regression analysis indicated that adverse childhood experience had a positive indirect effect on symptom  severity, mediated by alexithymia | Large sample size  Not video-EEG confirmed  FND sample not consecutively recruited |
| Kienle et al.,  2018 | FND, multiple (19)  HC (19)  Mean age 42.7  F:M ratio 2.16:1 Inpatient setting | Intervention  Mixed psychotherapy and physiotherapy  SDQ-20 was used as a marker of symptom severity  Self-reported Likert-scale severity measure, alexithymia also measured  EEG used to measure the cortical correlates of emotional  regulation | SDQ-20 | SDQ-20 measures of central tendency were not reported for groups  They reported a slight decrease in SDQ-20 score from baseline following treatment; this did not reach statistical significance  No change in subjective symptom report or alexithymia/emotion regulation pre and post treatment | Small sample may have prevented group differences  FND sample not consecutively recruited  Did not exclude active severe psychiatric comorbidity |
| Hammond- Tooke et al., 2018 | FND; mixed sample (n = 29); HC (n = 29)  FND F:M Gender ratio 2.22:1  Mean age 43.9  Outpatient setting | Case/Control  Response inhibition in FND patients tested via go/no-go task  Dissociation scores | DES | FND patients made more errors on go/no-go tasks | Did not exclude severe psychiatric comorbidity  Comorbid neurological disorder not excluded explicitly  Possible confounding effect of medications not assessed  FND sample not consecutively  recruited |
| Boesten et al., 2018 | FND, FND-Seizures –  traumatized group (n = 148); FND, FND-Seizures –  non-traumatised group (n = 69)  FND-Seizures-traumatised F:M gender ratio 6.4:1;  average age 38.65  FND-Seizures-non- traumatised F:M ratio 3.6:1; average  age 38.04  Outpatient setting | Cohort study  Impact of trauma on FND-Seizures severity and presentation  Quality of life in epilepsy, QOLIE | TSI | Mean QOLIE total was significantly less in traumatised FND-Seizures patients  Mean TSI- Dissociation was higher in traumatised FND-Seizures patients; p =0.032 | FND diagnosis not explicitly made by specialist  Did not exclude active severe psychiatric comorbidity  Medication effects considered |
| Perez et al., 2018 | FND, mixed (n = 26); HC (n = 27)  FND F:M Gender ratio 4.2:1  Mean age 40.3  Outpatient setting | Case/Control  Cortical and subcortical thickness in FND and controls, related to levels of dissociation  Association with trauma | SDQ- 20  DES | Patients with high levels of somatoform dissociation (SDQ>35) showed reduced left caudal anterior cingulate cortical (ACC) thickness compared to controls  SDQ-20 inversely correlated with ACC thickness  No significant statistical correlation between SDQ or DES score and levels of abuse | Measured psychoform and somatoform dissociation  Control group not matched for age and gender  FND sample not consecutively recruited |
| del Río- Casanova et al., 2018 | FND, unspecified (n  = 43); HC (n = 42)  FND F:M Gender ratio 7.6:1  Outpatient setting | Case/Control  Emotional regulation in FND  Dissociation scores  Emotional regulation | DES  SDQ-20 | Psychoform dissociation significantly correlated to emotional dysregulation (r=0.309)  Somatoform dissociation significantly associated to emotional dysregulation (r=0.324) and anxiety (r=0.301) | Measured psychoform and somatoform dissociation  did not exclude history of migraine as possible confounder  Possible confounding effect of medications not assessed  FND sample not consecutively recruited |
| Akyüz et al., 2017 | FND, unspecified (n  = 60)  Female patients Mean age 36.27 Outpatient setting | Cohort study  Socio-demo- graphic and clinical characteristics comorbidity, childhood traumatic experiences in FND patients  Comorbid dissociative disorders | DES | Dissociative disorder was comorbid in 48.3%  There were significant positive correlations between DES score and several trauma scores e.g. Physical abuse (r = 0.46; p = 0.001), Sexual abuse (r = 0.395; p = 0.006) | No control group  FND sample not consecutively recruited  Possible confounding effect of medications not assessed |
| Martino et al., 2017 | FND, FND-Seizures (10)  MDD (10)  Female  Mean age 38.22 Outpatient setting | Case / control  MDD vs FND  Wide battery of psychopathologic al tests  Dissociation and somatization | DES  SDQ-20 | Significant differences were seen in alexithymia, anxiety, somatoform dissociation and somatization; all greater in FND > MDD  SDQ-20 and DES  scores were greater in FND patients compared to MDD; this was only statistically significant for SDQ- 20 | Psychoform and somatoform dissociation were measured  Sample size of the groups was very small  Patients were consecutively recruited |
| Kienle et al.,  2017 | FND, multiple (60)  PTSD (39)  HC (40)  Mean age 42.6  F:M 3:1  Unclear setting | Case / Control  PTSD vs FND  DES, SDQ-20  used  PTSD diagnostic scale  Trauma history and alexithymia measured | DES  SDQ-20 | 20 of the 60 FND patients met diagnostic criteria for PTSD  The PTSD/FND  subgroup endorsed higher SDQ-20 and DES scores than the FND alone group  PTSD patients reported the highest DES scores; SDQ-20 scores were comparable to the DD/PTSD subgroup  History of traumatic experiences and severity of PTSD symptoms explained 30% of the variance in FND SDQ-20  scores  Alexithymia varied with DES (r=.4, p = 0.001) and SDQ (r=0.3, p = 0.02) | Good sample size  Measured psychoform and somatoform dissociation FND sample not consecutively recruited  Did not exclude active severe psychiatric comorbidity |
| Ekanayake et al., 2017 | FND, FND-motor (n = 59); FND, FND-Seizures (n = 43); HC (n = 26)  FND-Seizures F:M gender ratio 2.69: 1  FMS F:M gender ratio 5.14:1  Mean age 40.5  Outpatient setting | Case/Control  Personality traits in FND; personality inventory  Emotional disturbance  Trauma  Differences between FND subtypes | DES | FND-seizures endorsed higher sexual abuse scores, alexithymia, dissociation scores, and overall psychopathology relative to FND-motor and HC | Control groups not age- and sex- matched  Possible confounding effect of medications not assessed  FND sample not consecutively recruited |
| Demartini et al., 2017 | FND, FND-motor (n = 20);  Anorexia nervosa (AN) (n = 20); HC (n  = 20)  FND F:M Gender ratio 2.33:1  Mean age 45.75 Outpatient setting | Case/Control  Comparing clinical characteristics between AN and FND patients  Trauma history  Emotional regulation | DES | AN and FND patients endorse significantly greater degrees of alexithymia relative to controls  DES score was greater in AN than FND; not significant  Similar levels of abuse reported between AN and FND | Small sample size  Did not exclude severe psychiatric comorbidity  Possible confounding effect of medications not assessed  Control group not matched for age and gender |
| Pick, Mellers & Goldstein, 2017 | FND, FND-Seizures (n = 40); HC (n = 43)  FND F:M Gender ratio 4:1  Outpatient setting | Case/Control  Dissociative experiences in FND-Seizures  Post-traumatic symptoms in FND-Seizures  Trauma history in FND-Seizures | MDI  SDQ-20 | Significantly higher sexual and physical abuse history in FND-Seizures relative to HC  Significantly greater PTSD symptoms in FND-Seizures relative to HC  66.7% of FND-Seizures met criteria for PTSD  Positive correlation between TEC-sexual abuse and psychoform dissociation  Positive correlation between psychoform dissociation and PTSD symptoms  MDI-depersonalisation positively correlated with severity of ictal symptoms  SDQ scores not significantly associated with seizure severity  Multivariate regression analysis revealed that sexual abuse history associated with a 3- fold increase in likelihood of being diagnosed with FND-Seizures | FND sample not consecutively recruited  Measured psychoform and somatoform dissociation  Effects of medications considered in group |
| Gonzalez- Vazquez et al., 2017 | FND, unspecified (n  = 38); Dissociative disorders (DD) (n = 30); Other Psych (n  = 292)  Mean age 39.44 Outpatient setting | Validity study  SDQ-20 scores in dissociative disorders, FND, and general psychiatric illnesses  Trauma history | DES  SDQ-20 | SDQ-20 scores were significantly higher in women than men  SDQ-20 scores significantly higher in dissociative disorders than in CD  SDQ-20 significantly correlated with trauma score in CD (r = 0.32, p <0.01)  Suggest DES cut-off score of 29.5 for CD diagnosis, 27.5 for DD; 81.6% sensitive, 71% specificity | Comorbid neurological disorder not excluded explicitly  Possible confounding effect of medications not assessed  Controls not age and sex matched  Measured psychoform and somatoform dissociation  FND sample not consecutively recruited |
| Myers et al., 2017 | FND, FND-Seizures (n = 148);  female n = 97; male n = 51  Mean age (female) 37  Mean age (male) 34.35  Outpatient setting | Cohort  Trauma history, avoidance behaviour, and dissociation in FND-Seizures  Compared between genders | TSI | Significantly higher prevalence of sexual abuse history in females than men, 42% vs 16%; p = 0.007  Significantly higher dissociation scores in women compared to men, p = 0.012 | No non-FND control group  Comorbid neurological disorder not excluded explicitly  FND sample not consecutively recruited |
| Demartini et al., 2016 | FND, FND-motor (n = 20); FND, FND-Seizures (n = 20); HC (n = 20)  FMS F:M gender ratio 5.66; average  age 45.7  FND-Seizures F:M gender ratio 3:1; average age  45.9  Outpatient setting | Dissociation in FND subtypes  Emotional dysregulation and psychopathology in FND | DES  SDQ-20  CDS | SDQ-20 score greater in FND-motor than FND-seizures  Mean DES score greater in FND-seizures than in FND-motor  Detachment (as measured by CDS) greater in FND-Seizures than FMS (p = 0.007) | Did not exclude severe psychiatric comorbidity  Possible confounding effect of medications not assessed  Measured psychoform and somatoform dissociation |
| Sarisoy et al., 2015 | FND, mixed sample (n = 60); HC (n = 60)  FND F:M gender ratio 9:1  Mean age 33.6 Outpatient setting | Case/Control  Temperament and character traits in FND relative to dissociative symptoms  Trauma history  FND was divided into two subgroups based on high or low dissociation as per DES (cut-off  >30) | DES | High harm avoidance, low self- directedness may be associated with pathological dissociation in conversion disorder patients | Comorbid neurological disorder was not excluded explicitly  Possible confounding effect of medications not assessed  FND sample not consecutively recruited |
| van der Hoeven et al., 2015 | FND, FND-motor (n = 55);  Neurological movement disorder (MD; n = 34); HC (n  = 52)  FND F:M gender ratio 1.68:1  Mean age 50.4 Outpatient setting | Case/Control  General psychopathology and dissociation in FND vs MD  Symptom Checklist (SCL-90- R) | DIS-Q  SDQ-20 | SCL-90-R score correlated with DIS-Q (r = 0.57; p = 0.001) and SDQ-20 (r = 0.37; p = 0.008)  39% of the FND group scored normally on all psychometric measures | Did not exclude severe psychiatric comorbidity  Possible confounding effect of medications not assessed  Measured psychoform and somatoform dissociation  FND sample not consecutively recruited |
| Steffen et al., 2015 | FND, mixed (n = 30); FND,  sensory/motor (n = 15); HC (n = 45)  FND F:M gender ratio 2.46:1  Mean age 40.4 Inpatient setting | Case/Control  Adverse childhood experiences in FND  SCL-90  Emotional regulation | SDQ-20 | SDQ-20 scores were higher in FND-mixed presentation participants  SDQ-20 score was positively correlated with alexithymia score  positive correlation between emotional adverse child events and SDQ-20 score | Controls not age and sex matched  Did not exclude severe psychiatric comorbidity  FND sample not consecutively recruited  Possible confounding effect of medications not assessed |
| O’Brien et al., 2015 | FND, FND-Seizures (n = 19); HC (n = 19)  FND-Seizures F:M gender ratio 2.1:1  Mean age 30 Outpatient setting | Case/Control  Psychopathology in FND-Seizures  Alexithymia | DES | DES score was significantly associated with frequency of seizures | Small sample size  Possible confounding effect of medications not assessed  FND sample not consecutively recruited |
| Stins et al.,  2015 | FND, unspecified (12)  HC (12)  Mean age 46  F:M 1.4:1  Outpatient setting | Case / control  Postural steadiness in FND  Maintenance of balance under varying conditions: eyes closed, eyes open, while performing a cognitive task | CADSS | Significantly more dissociative symptoms than controls (p=0.05)  Dissociation correlated significantly between measures of postural sway from the centre of the platform FND participants exhibited more postural instability than controls  Addition of cognitive task improved postural steadiness | Very small patient sample  Incomplete CADSS used  FND sample not consecutively recruited |
| Yayla et al.,  2015 | FND, unspecified (n  = 54)  FND F:M gender ratio 5.4:1  Mean age 28.05 Inpatient setting | Cohort study  Dissociative disorder comorbidity  DES scores in FND and FND comorbid with DD | DES | 37.03% of patients had comorbid DD  DD-NOS (18.52%, n =10), dissociative amnesia (14.81%, n = 8) and dissociative depersonalisation disorder (1.08%, n = 2)  Mean DES significantly higher in FND with comorbid DD; 29.3 vs. 9.11, p < .001 | No control group  Possible confounding effect of medications not assessed |
| Cohen et al.,  2014 | FND, FND-Seizures (n = 46)  FND-Seizures F:M gender ratio 5.57:1  Mean age 41.8 Outpatient setting | Cohort study  Investigate possible overlap between dissociation and other psychopathology | DES | Higher DES score predicted by greater distress, secondary to depression, somatic concerns  Depression and anxiety strongly correlated with DES score  70.2% of the variance associated with DES score was explained by psychological distress  and locus of control | No control group  FND diagnosis not explicitly made by specialist  Possible confounding effect of medications not assessed |
| van der Kruijs et al., 2013 | FND, FND-Seizures (n = 21); HC (n = 27)  FND-Seizures F:M gender ratio 1.63:1  Mean age 34 Outpatient setting | Case/Control  Resting-state fMRI  Dissociative scores | SDQ-20  DIS-Q  DES | Significant association between abnormal default mode network connectivity and level of dissociation | Small sample size  FND-Seizures diagnosis not explicitly confirmed by video-EEG  Measured psychoform and somatoform dissociation  FND sample not consecutively recruited  Considered medication impact on groups |
| Güleç et al., 2013 | FND, multiple (94); previous suicide attempt (33), no previous suicide attempt (61)  HC (30)  FND-suicide attempt F:M gender ratio 5.6:1;  mean age 30.3  FND no suicide attempt F:M gender ratio 5.77;  Mean age 30.82  Outpatient setting | Case/control  Levels of dissociation in FND participants with or without suicide attempt history  Emotional regulation, psychopathology  Trauma history | DES | Suicide attempt group had higher mean DES scores  Elevated risky alcohol use in suicide attempters | Large sample size  Possible confounding effect of medications not assessed |
| Xue et al.,  2013 | FND, FND-Seizures (15)  HC (15)  F:M ratio 1.11:1  Mean age 20.5 Outpatient setting | Case/control  EEG in FND and HC groups  Dissociation scores | SDQ-20 | Altered connectivity between different brain regions  Not correlated with SDQ score | Small sample size  FND sample not consecutively recruited  Considered medications |
| Scévola et al., 2013 | FND, FND-Seizures (35)  ES (49)  Mean age 37.54  F:M ratio 3.38:1 Outpatient setting | Case / control  Psychiatric comorbidity in FND-Seizures and ES  Trauma history |  | Comorbid DD found in 37.14% of FND patients  Depression rate was comparable in the two groups  Anxiety, PD and DD were more prevalent in FND-Seizures than ES  Trauma history was significantly more frequent in FND-Seizures than  ES | FND sample not consecutively recruited  Did not exclude active severe psychiatric comorbidity |
| Mitchell et al., 2012 | FND, FND-Seizures (39)  Mean age 41.6  F:M ratio 2.25:1 Outpatient setting | Cohort  Dissociation and quality of life in FND-Seizures  Other psychopathologic al scoring systems | DES | 36.7% of the sample scored higher than 30 on the DES  Median DES score 20.7 (IQR 30.4)  Quality of life (QOLIE-31 score) significantly and  negatively correlated with DES score (r=- 0.64, p <0.001); this remained significant when controlling for depression, anxiety and other psychiatric  comorbidities | Lack of a control group  Did not exclude active severe psychiatric comorbidity |
| Brown et al.,  2013 | FND, FND-Seizures (43)  ES (24)  F:M ratio 1.86:1 Outpatient setting | Case / control  Emotional regulation and dissociation in FND and ES | SDQ-20 | Subgroup of FND-  Seizures patients described with high levels of emotional dysregulation and alexithymia  SDQ-20 scores were  higher in this group | Possible confounding effect of medications not assessed  Did not exclude severe psychiatric comorbidity |
| Myers et al.,  2012 | FND, FND-Seizures (66)  ES (35)  F:M ratio 10:1  Mean age 38.4 Outpatient setting | Case/Control  Exploring factors that predict FND-Seizures rather than ES  Emotional dysregulation, dissociation | TSI | Significant association between alexithymia and dissociation score | Large sample size  Control group not matched for age and gender  Active severe psychiatric comorbidity  excluded |
| Van der Kruijs et al., 2012 | FND, FND-Seizures (11)  HC (12)  F:M ratio 1.2  Mean age 34 Outpatient setting | Case / control  Functional connectivity in FND  Dissociation scores  Neurophysiologic al correlates of FND-Seizures | DIS-Q  DES  SDQ-20 | Stronger functional connectivity in insula, inferior frontal gyrus, parietal cortex, and precentral sulcus correlate to DISQ, DES and SDQ scores  Lower cognitive performance in FND-Seizures group | Small sample size  Measured psychoform and somatoform dissociation  FND sample not consecutively recruited  Active severe psychiatric comorbidity excluded |
| Kranick et al., 2011 | FND-motor (64)  HC (34)  Hand dystonia (39)  F:M ratio 2.55:1  Mean age 45.2 Outpatient setting | Case / control  Trauma history, personality and psychopathology including dissociation | DES | No significant difference on dissociation scores observed  Greater history of trauma in FMS group | Did not measure somatoform dissociation  FND sample not consecutively recruited  Did not exclude active severe psychiatric comorbidity  Large FND sample  size |
| Proenca et al., 2011 | FND, FND-Seizures (20)  ES (20)  F:M ratio 3:1 Outpatient setting | Case / control  FND-Seizures vs temporal lobe epilepsy  Trauma history  Dissociation | DES | DES score significantly higher in FND-Seizures vs ES (54.4 ± 23.2; 22 ± 16.4; p = 0.001)  Trauma history more severe in FND-Seizures vs ES | Small sample size  FND sample not consecutively recruited  Did not exclude active severe psychiatric comorbidity |
| Marchetti et al., 2009 | FND, FND-Seizures (13)  F:M ratio 12:1  Mean age 36 Mixed setting | Cohort  DD comorbidity in FND |  | 7.69% of sample found to have comorbid DD (1 patient)  DDNOS diagnosed | Validated diagnostic tool used  Small sample size  Lack of control group  FND sample not consecutively recruited  Control group not matched for age and gender  Did not exclude active severe psychiatric comorbidity |
| Ozcetin et al., 2009 | FND, FND-Seizures (56)  HC (59)  Female sample Mean age 33.75 Outpatient setting | Case / control  Dissociation in FND-Seizures  Trauma history  Correlations between trauma and dissociation | DIS-Q | 75% of patients DIS- Q > 2.5  Trauma measure strongly correlated with DISQ score (Spearman’s r 0.87; p <0.01) | May have benefitted from administering SCID  /DDIS to diagnose DD  FND sample not consecutively recruited  Control group not matched for age and gender  Active severe psychiatric comorbidity  excluded |
| Espirito- Santo et al., 2009 | FND, Multiple (26)  DD (38)  Somatization disorder (40)  F:M ratio 3.33:1  Mean age 27.4 Mixed setting | Case / control  Dissociative scores in different, similar conditions | DES  SDQ-20 | 78% of FND group above DES cut-off 59.5% above SDQ-20 cut-off  Mean scores were greater in FND than DD, somatization disorders and HC | Investigating multiple different patient types  Active severe psychiatric comorbidity excluded |
| Reedijk et al., 2008 | FND, unspecified (26)  Complex Regional Pain Syndrome, CRPS (46)  Affective disorders (50)  F:M ratio 5:1  Mean age 38 Outpatient setting | Case / Control  Dissociation levels  Psychopathology  Trauma history | DES  SDQ-20 | DES was higher in FND group; SDQ-20 was equal in FND and CRPS  Similar levels of trauma in FND and CRPS group | Multiple patient groups  Psychoform and somatoform dissociation screened  FND sample not consecutively recruited  Did not exclude active severe psychiatric comorbidity |
| Kuyk et al.,  2007 | FND, FND-Seizures (26)  F:M gender ratio 3.4:1  Mean age 30.6 Inpatient setting | Intervention  Psychotherapy and psychomotor rehabilitation therapy  Psychopathology and dissociation scores at induction, end of treatment and follow up | DIS-Q | Seizure frequency diminished, lasting at 6 months post treatment  Mean DIS-Q scores reduced between start of treatment (T1), end of treatment (T2) and follow-up (T3):  T1 – T2: 1.86 vs 1.69 (p = 0.05)  T2 – T3: 1.69 vs 1.48 (p = 0.42)  T1 – T3: 1.86 vs 1.48 (p=0.01) | No control group  Not blinded or randomized  FND sample not consecutively recruited  Did not exclude active severe psychiatric comorbidity |
| Bodde et al.,  2007 | FND, FND-Seizures (22)  F:M gender ratio 6.33:1  Mean age 30.4 Outpatient setting | Cohort study  DIS-Q subscale scores  SCL-90-R  Personality traits  Psychopathology and dissociation at diagnosis and then follow-up 4-  6 years later | DIS-Q  sub- scale | All mean DIS-Q subscale scores (subscales 1 – 4) were reduced from their baseline at re-evaluation; three of these reductions were statistically significant  DIS-Q was not significantly correlated to seizure  frequency | No control group  Appropriate duration of follow- up  FND sample not consecutively recruited  Did not exclude active severe psychiatric comorbidity |
| Evren & Can, 2007 | FND, multiple (55) Male sample Mean age 21 Mixed setting | cohort study  Male soldiers with FND  Dissociation levels  Emotional dysregulation | DES | DES was positively correlated with duration of military service  DES positively correlated with alexithymia, r = 0.44  Patients with motor symptoms scored lower on the DES, whilst patients with  seizures scored higher | Lack of control group  FND sample not consecutively recruited  Sample was only males completing military service  Excluded active severe psychiatric comorbidity |
| Espirito- Santo et al., 2006 | FND, multiple (25)  DD, (36)  PTSD (49)  Various psych. (116)  Outpatient setting | Case/control  Validity study  Somatoform dissociation in different patient populations | SDQ-20 | FND, DD and PTSD  groups reported similar levels of somatoform dissociation  Cut-off score of 35 suggested for best sensitivity/specificity | FND sample small  Did not exclude active severe psychiatric comorbidity |
| Goldstein & Mellers, 2006 | FND, FND-Seizures (25)  Partial ES (19)  F:M Gender ratio 3.17:1  Mean age 35.52 Outpatient setting | Case / control  Dissociation and other psychopathology in FND-Seizures and ES | DES | 9 patients scored >30 on DES | FND sample highly comorbid with psychiatric disease  FND sample not consecutively recruited |
| Spinhoven  et al., 2004 | FND, FND-Seizures (61)  Mean age 31.5 F:M gender ratio 3.36:1  FND, unspecified (102; 54)  Chronic pelvic pain (52)  Mixed setting | Case / Control  Trauma history  Dissociation profile  Psychoform and somatoform dissociation measures  Comparing unspecified FND, FND-Seizures, and chronic pelvic pain groups | DES  SDQ-20  DIS-Q | Mean DES scores for unspecified FND groups were comparable to the general population  History of abuse was more common in chronic pelvic pain group than FND subtypes  Psychopathology and history of abuse were weakly correlated with SDQ- 20 score in FND groups | Large sample size  Use of psychoform and somatoform dissociation measures  Not video-EEG confirmed  Control group not matched for age and gender  Did not exclude active severe psychiatric comorbidity |
| Akyüz et al.,  2004 | FND, FND-Seizures (33)  ES (30)  Female  Mean age 27.7 Unclear setting | Case / control  FND-Seizures vs ES  Dissociation  Trauma history  Psychopathology; anxiety | DES  CADSS | ES CADSS score greater in ES than FND-Seizures, not significant; DES score significantly higher in FND-Seizures than ES  Abuse rates in FND are high  Suicide attempts higher in FND than ES | Correlations between suicide rates and psychopathology would have been of interest  Use of more than one dissociative scale  Females only  FND sample not consecutively recruited |
| Guz et al.,  2004 | FND, multiple (87) Somatization disorder (71)  F:M ratio 5.21:1  Mean age 37.5 Mixed setting | Case / control  Somatization disorder vs FND  Psychopathology and dissociation  SCL-90-R  Suicide ideation scale | DES | No appreciable differences in DES, psychopathology or suicidal ideation between the two groups | FND sample not consecutively recruited  Controls were age and sex matched  Large sample size |
| Baillés et al., 2004 | FND, FND-Seizures (30)  F:M gender ratio 9:1  Mean age 34.1  Inpatient setting | Cohort  Abuse and trauma history  DD comorbidity |  | 50% of the sample had comorbid DD  The most frequent comorbid DD was DDNOS and abuse rates were high in this population | No control group  Only FND sample not consecutively recruited  Did not exclude active severe psychiatric comorbidity |
| Reuber et al., 2003 (i) | FND, FND-Seizures (98)  ES (63)  F:M gender ratio 4.44:1  Mixed setting | Case / Control  Compares seizure semiology in FND-Seizures and ES  Psychopathology and dissociation in FND-Seizures and ES  Correlations with seizure severity  SCL-90-R | DES | High seizure severity associated with high DES score  Strong positive correlation between DES and general psychopathology (r=0.66, p = 0.01)  FND-seizures were better identified by somatization score, not dissociation score, in a linear regression analysis  DES did not contribute independently to discrimination of FND-seizures from epilepsy patients | Large sample size included  Did not exclude active severe psychiatric comorbidity |
| Reuber et al., 2003 (ii) | FND, FND-Seizures- Status group (33)  FND, FND-Seizures – no status group (52)  ES (64)  F:M gender ratio 5.6:1  Outpatient setting | Case / control  Seizure severity  Dissociative symptoms  Personality questionnaires | DES | Mean DES significantly higher in FND-seizure patients with non-epileptic status vs those with non-epileptic status (p = 0.001) | Large sample size  FND sample not consecutively recruited  Control group not matched for age and gender  Did not exclude active severe psychiatric comorbidity |
| Guz et al.,  2003 | FND, FND-motor (24);  mean age 36; F:M 6:1  FND, FND-Sensory (5); mean  age 48  FND, Mix (43);  mean age 36; F:M 7.6:1  FND, FND-Seizures (23); mean age 36; F:M 4.75:1  Mixed setting | Cohort  FND subtype dissociation scores  Comparing subtypes in terms of DES score and demographic features | DES | Suggest that the FND-  Seizures subtype be classified as a ‘dissociative disorder,’ whereas the motor subtype as a somatoform disorder  Motor subtype – least patients with a DES > 30; FND-Seizures had the most | Large sample of FND patients  Comparisons between the different subtypes with respect to psychoform dissociation  FND sample not consecutively recruited |
| Tezcan et al., 2003 | Multiple; all comorbid DD; (18) Multiple; no comorbid DD; (17)  Mix (7)  FND-motor (5)  FND-Seizures (26)  FND-sensory (21)  Mean age 27.67  F:M 8:1  Inpatient setting | Cohort  Comparing dissociation in different FND subtypes  59 FND patients overall of different subtypes  Split group into DD-comorbid group and one without comorbid DD  Trauma and  abuse history | DES | Highest DES in FND with comorbid DD  Most comorbid DD was DID | Used SCID-D  Not video-EEG confirmed  Did not exclude active severe psychiatric comorbidity |
| Roelofs et al., 2002 (i) | FND, multiple (50)  Affective disorders, (50)  F:M 5.25:1  Mixed setting | Cohort  DD comorbidity in FND  Hypnotisability and dissociation in FND and in affective disorders | DES  SDQ-20  DIS-Q | Hypnotic suggestibility was positively correlated with number of functional neurological symptoms  SDQ-20 was positively correlated with number of functional neurological symptoms, r = 0.39, p  <0.01  DES and SDQ-20  scores were greater in FND; DIS-Q scores were the same between groups.  Only SDQ-20 score difference was statistically significant | Measures of psychoform and somatoform dissociation were used  FND sample not consecutively recruited  Did not exclude active severe psychiatric comorbidity |
| Roelofs et al., 2002 (ii) | FND, multiple (54)  Affective disorders (50)  Same population of FND participants as above  Mixed setting | Case / control  Affective disorders group vs FND group  Rates of child abuse  Dissociative scores  Same population as the one above. | DES  SDQ-20 | Higher incidence of childhood abuse in FND compared to affective disorder group  15% of FND patients did not report any childhood abuse  DES and SDQ-20  scores for patients displaying varying degrees of trauma  positive maternal dysfunction history;(34) DES 14.5 ± 13.9; SDQ-20 32.7 ± 8.7  negative maternal dysfunction history;  (20) DES 7.6 ± 7.1; SDQ20 27.3 ± 6.2 positive paternal dysfunction history;  (27) DES 11.4 ± 10.5; SDQ20 30.4 ± 6.9  negative paternal dysfunction history; (27) DES 12.5 ± 11.4;  SDQ20 30.9 ± 9.5  positive PA history; (15) DES 13.8 ± 9.4;  SDQ20 34.1 ± 8.3  negative PA history ; (39) DES 11.2 ± 11.4;  SDQ20 positive SA history; (13) DES 12.3 ± 7.7;  SDQ20 31.2 ± 7.5  negative SA; (41) DES 11.8 ± 11.8; SDQ20  30.6 ± 8.5  multiple traumas;  (21) DES 13.6 ± 8.7; SDQ20 34.6 ± 7.9  no multiple traumas; (25) DES 11.9 ± 13.1;  SDQ20 28.8 ± 7.829.4 ± 7.9 | Sample presented with many psychiatric comorbidities  FND sample not consecutively recruited  Did not exclude active severe psychiatric comorbidity |
| Moene et al., 2001 | FND, unspecified (102)  HC (89)  Mixed psych. (278)  Mean age 39.1  F:M ratio 3.08: 1 Mixed setting | Case / control  Hypnotisability, trauma history and dissociation  SCL-90-R  Comorbid psychiatric disorders  Inpatient and outpatient FND sample differences. | DIS-Q | 10 patients had comorbid dissociative disorder as per DSM criteria  Inpatients with FND scored significantly higher on DIS-Q than outpatient sample (1.8 v 1.5, p <0.01)  DIS-Q score was higher in the mixed psychiatric group than the FND combined group; both were greater than the normal control group  DIS-Q and psychopathology was significantly higher in traumatised FND participants opposed to  non-traumatised | Very large sample size  Results pertaining to inpatient and outpatients  Control group not matched for age and gender  Non-respondent data is described |
| Litwin & Cardeña, 2000 | FND, FND-Seizures (10)  ES (31)  Mean age 30.5 Female Inpatient setting | Case / control  FND-Seizures v ES  Hypnotisability, dissociative tendency in ES and FND-Seizures patients | DES | Comorbid DD in 80% of FND participants  Patient demographics and seizure semiology were good predictors of group membership, dissociation and  hypnotisability were not | Very small FND sample size  FND sample not consecutively recruited  Control group not matched for age and gender  Did not exclude active severe psychiatric comorbidity |
| Goldstein et al., 2000 | FND, FND-Seizures (20)  HC (20)  F:M 4:1  Mean age 34.35 Outpatient setting | Case / control  Characteristics of FND-Seizures patients  Dissociation; DES, Perceptual alteration scale (PAS)  Emotional coping in FND  Hypnotisability  Psychopathology (depressive and anxiety symptoms) | DES  PAS | FND participants scored higher in dissociative scales relative to controls  PAS score was not statistically significantly greater than control group  Hypnotisability measures were higher in the control group  Coping style in FND-Seizures was predominantly escape-avoidance with less planful  problem solving | Multiple scales of psychoform dissociation used  FND sample not consecutively recruited  Control group not matched for age and gender |
| Spitzer et al., 1999 | FND, FND-Seizures (21); FND-motor (16); FND-sensory (15); FND-Mixed  (20); total (72)  General psych. patients (96)  F:M 3:1  Mean age 33 Inpatient setting | Case / Control  Dissociative and general psychopathologic al profile of differing subtypes of FND  DES  SCL-90-R | DES | No difference in SCL- 90-R between the two groups  Dissociative symptoms significantly greater in FND than controls | Only measured psychoform dissociation  Large sample size  Differentiated scores for different FND subtypes  FND sample not consecutively recruited  Did not exclude active severe psychiatric comorbidity |
| Wood et al.,  1998 | FND, FND-Seizures (9)  ES (9)  Outpatient setting | Case / control  FND-Seizures vs ES  Families of patients examined as well | DES | DES does not differentiate between ES and FND-Seizures | Very small sample size  FND sample not consecutively recruited  Control group not matched for age and gender  Examined psychological profiles of relatives in addition to patients  Did not exclude active severe psychiatric comorbidity |
| Alper et al.,  1997 | FND, FND-Seizures (132)  ES (169)  Mean age 32.78  F:M ratio 2.3:1 Inpatient setting | Case / control  FND-Seizures vs complex partial epilepsy  DES administered  Principal components analysis | DES | Mean DES score 15.1 in FND-Seizures; 12.7 in ES -  difference was not significant (p=0.079)  Depersonalisation- derealization and absorption DES sub scores were better indicators of  FND-Seizures vs ES | Very large sample size  Excluded active severe psychiatric comorbidity  Compared with controls |

### Supplementary Table 4: Newcastle Ottawa Scale Ratings for Case-control Studies

| Newcastle Ottawa Scale Ratings for Case-control Studies | | | | | | | | | | |
| --- | --- | --- | --- | --- | --- | --- | --- | --- | --- | --- |
|  | Selection | | | | Comparability | | Exposure | | | Total Score (0-9) |
|  | Is the case definition adequate? | Representativeness of the cases | Selection of Controls | Definition of Controls | Comparability of cases and controls on the basis of the design or analysis (Age) | Comparability of cases and controls on the basis of the design or analysis (Other factors) | Ascertainment of exposure | Same method of ascertainment for cases and controls | Non-Response rate |  |
| Hammond-Tooke et al., 2018 | a) yes with independent validation * | a) consecutive or obviously representative series of cases * | a) community controls * | a) no history of disease (endpoint)* | a) study controls for age * | b) study controls for any additional factor * | a) secure record (e.g. surgical records) * | a) yes * | unable to calculate | 8 |
| del Río-Casanova et al., 2018 | a) yes with independent validation * | a) consecutive or obviously representative series of cases * | a) community controls * | a) no history of disease (endpoint) * | a) study controls for age * | b) study controls for any additional factor * | a) secure record (e.g. surgical records) * | a) yes * | unable to calculate | 8 |
| Perez et al., 2018 | a) yes with independent validation * | b) potential for selection biases or not stated | a) community controls * | a) no history of disease (endpoint) * | Did not control age | b) study controls for any additional factor * | a) secure record (e.g. surgical records) * | a) yes * | unable to calculate | 6 |
| Ekanayake et al., 2017 | a) yes with independent validation * | a) consecutive or obviously representative series of cases * | b) hospital controls | a) no history of disease (endpoint) * | Did not control age | b) study controls for any additional factor * | a) secure record (e.g. surgical records) * | b) no | b) non-respondents described | 5 |
| Demartini et al., 2017 | a) yes with independent validation * | b) potential for selection biases or not stated | a) community controls * | a) no history of disease (endpoint) * | No discussion of potential confounders/controls | No discussion of potential confounders/controls | a) secure record (e.g. surgical records) * | a) yes * | unable to calculate | 5 |
| González-Vázquez et al., 2017 | b) yes e.g. record linkage or based on self reports | b) potential for selection biases or not stated | b) hospital controls | b) no description of source | No discussion of potential confounders/controls | No discussion of potential confounders/controls | a) secure record (e.g. surgical records) * | a) yes * | unable to calculate | 2 |
| Pick, Mellers & Goldstein, 2017 | a) yes with independent validation * | a) consecutive or obviously representative series of cases * | a) community controls * | a) no history of disease (endpoint) * | a) study controls for age * | b) study controls for any additional factor * | a) secure record (e.g. surgical records) * | a) yes * | unable to calculate | 8 |
| Demartini et al., 2016 | a) yes with independent validation * | a) consecutive or obviously representative series of cases * | a) community controls * | a) no history of disease (endpoint) * | a) study controls for age * | b) study controls for any additional factor * | a) secure record (e.g. surgical records) * | a) yes * | unable to calculate | 8 |
| Myers et al., 2019 | a) yes with independent validation * | a) consecutive or obviously representative series of cases * | b) hospital controls | a) no history of disease (endpoint) * | No discussion of potential confounders/controls | No discussion of potential confounders/controls | a) secure record (e.g. surgical records) * | a) yes * | a) same rate for both groups * | 6 |
| Sarisoy et al., 2015 | b) yes e.g. record linkage or based on self reports | a) consecutive or obviously representative series of cases * | a) community controls * | a) no history of disease (endpoint)* | a) study controls for age * | b) study controls for any additional factor * | a) secure record (e.g. surgical records) * | a) yes * | unable to calculate | 7 |
| van der Hoeven et al., 2015 | b) yes e.g. record linkage or based on self reports | a) consecutive or obviously representative series of cases * | a) community controls * | a) no history of disease (endpoint) * | Did not control age | b) study controls for any additional factor * | a) secure record (e.g. surgical records) * | a) yes * | unable to calculate | 6 |
| Steffen et al., 2015 | b) yes e.g. record linkage or based on self reports | b) potential for selection biases or not stated | a) community controls * | a) no history of disease (endpoint) * | Did not control age | No discussion of potential confounders/controls | a) secure record (e.g. surgical records) * | a) yes * | unable to calculate | 4 |
| O’Brien et al., 2014 | a) yes with independent validation * | a) consecutive or obviously representative series of cases * | a) community controls * | a) no history of disease (endpoint) * | a) study controls for age * | b) study controls for any additional factor * | a) secure record (e.g. surgical records) * | a) yes * | b) non-respondents described | 8 |
| van der Kruijs et al., 2013 | a) yes with independent validation * | b) potential for selection biases or not stated | a) community controls * | a) no history of disease (endpoint) * | No discussion of potential confounders/controls | No discussion of potential confounders/controls | a) secure record (e.g. surgical records) * | a) yes * | b) non-respondents described | 5 |
| Güleç et al., 2013 | a) yes with independent validation * | b) potential for selection biases or not stated | a) community controls * | a) no history of disease (endpoint) * | No discussion of potential confounders/controls | No discussion of potential confounders/controls | a) secure record (e.g. surgical records) * | a) yes * | unable to calculate | 5 |
| Xue et al., 2013 | a) yes with independent validation * | a) consecutive or obviously representative series of cases * | a) community controls * | a) no history of disease (endpoint) * | a) study controls for age * | b) study controls for any additional factor * | a) secure record (e.g. surgical records) * | a) yes * | unable to calculate | 8 |
| Brown et al., 2013 | a) yes with independent validation * | a) consecutive or obviously representative series of cases * | b) hospital controls | a) no history of disease (endpoint) * | Did not control age | No discussion of potential confounders/controls | a) secure record (e.g. surgical records) * | a) yes * | a) same rate for both groups * | 6 |
| Myers et al., 2013 | a) yes with independent validation * | a) consecutive or obviously representative series of cases * | b) hospital controls | a) no history of disease (endpoint) * | Did not control age | No discussion of potential confounders/controls | a) secure record (e.g. surgical records) * | a) yes * | b) non-respondents described | 5 |
| van der Kruijs et al., 2011 | a) yes with independent validation * | b) potential for selection biases or not stated | a) community controls * | a) no history of disease (endpoint) * | No discussion of potential confounders/controls | No discussion of potential confounders/controls | a) secure record (e.g. surgical records) * | a) yes * | b) non-respondents described | 5 |
| Kranick et al., 2011 | a) yes with independent validation * | a) consecutive or obviously representative series of cases * | a) community controls * | a) no history of disease (endpoint) * | a) study controls for age * | b) study controls for any additional factor * | a) secure record (e.g. surgical records) * | a) yes * | unable to calculate | 8 |
| Proenca et al., 2011 | a) yes with independent validation * | a) consecutive or obviously representative series of cases * | b) hospital controls | a) no history of disease (endpoint) * | a) study controls for age * | b) study controls for any additional factor * | a) secure record (e.g. surgical records) * | a) yes * | unable to calculate | 7 |
| Marchetti et al., 2009 | a) yes with independent validation * | a) consecutive or obviously representative series of cases * | b) hospital controls | a) no history of disease (endpoint) * | No discussion of potential confounders/controls | No discussion of potential confounders/controls | a) secure record (e.g. surgical records) * | a) yes * | c) rate different and no designation | 5 |
| Ozcetin et al., 2009 | a) yes with independent validation * | b) potential for selection biases or not stated | a) community controls * | a) no history of disease (endpoint) * | Did not control age | No discussion of potential confounders/controls | a) secure record (e.g. surgical records) * | a) yes * | b) non-respondents described | 5 |
| Espirito-Santo & Pio-Abreu, 2009 | a) yes with independent validation * | b) potential for selection biases or not stated | b) hospital controls | a) no history of disease (endpoint)* | No discussion of potential confounders/controls | No discussion of potential confounders/controls | a) secure record (e.g. surgical records) * | a) yes * | a) same rate for both groups * | 5 |
| Reedijk et al., 2008 | a) yes with independent validation * | a) consecutive or obviously representative series of cases * | b) hospital controls | a) no history of disease (endpoint) * | Did not control age | No discussion of potential confounders/controls | a) secure record (e.g. surgical records) * | a) yes * | b) non-respondents described | 5 |
| Espirito-Santo & Pio-Abreu, 2006 | a) yes with independent validation * | a) consecutive or obviously representative series of cases * | b) hospital controls | a) no history of disease (endpoint) * | No discussion of potential confounders/controls | No discussion of potential confounders/controls | a) secure record (e.g. surgical records) * | a) yes * | unable to calculate | 5 |
| Goldstein & Mellers, 2005 | a) yes with independent validation * | a) consecutive or obviously representative series of cases * | b) hospital controls | a) no history of disease (endpoint) * | Did not control age | No discussion of potential confounders/controls | a) secure record (e.g. surgical records) * | a) yes * | unable to calculate | 5 |
| Spinhoven et al., 2004 | a) yes with independent validation * | a) consecutive or obviously representative series of cases * | b) hospital controls | a) no history of disease (endpoint) * | No discussion of potential confounders/controls | No discussion of potential confounders/controls | a) secure record (e.g. surgical records) * | a) yes * | unable to calculate | 5 |
| Akyüz et al., 2004 | a) yes with independent validation * | b) potential for selection biases or not stated | b) hospital controls | a) no history of disease (endpoint) * | No discussion of potential confounders/controls | No discussion of potential confounders/controls | a) secure record (e.g. surgical records) * | a) yes * | a) same rate for both groups * | 5 |
| Guz et al., 2004 | a) yes with independent validation * | a) consecutive or obviously representative series of cases * | b) hospital controls | a) no history of disease (endpoint) * | No discussion of potential confounders/controls | No discussion of potential confounders/controls | a) secure record (e.g. surgical records) * | a) yes * | b) non-respondents described | 5 |
| Reuber et al., 2003 | a) yes with independent validation * | a) consecutive or obviously representative series of cases * | b) hospital controls | a) no history of disease (endpoint) * | Did not control age | b) study controls for any additional factor * | a) secure record (e.g. surgical records) * | a) yes * | a) same rate for both groups * | 7 |
| Reuber et al., 2003 | a) yes with independent validation * | b) potential for selection biases or not stated | b) hospital controls | a) no history of disease (endpoint) * | Did not control age | No discussion of potential confounders/controls | a) secure record (e.g. surgical records) * | a) yes * | b) non-respondents described | 4 |
| Roelofs et al., 2002 | a) yes with independent validation * | a) consecutive or obviously representative series of cases * | b) hospital controls | a) no history of disease (endpoint) * | a) study controls for age * | b) study controls for any additional factor * | a) secure record (e.g. surgical records) * | a) yes * | b) non-respondents described | 7 |
| Roelofs et al., 2002 | a) yes with independent validation * | a) consecutive or obviously representative series of cases * | b) hospital controls | a) no history of disease (endpoint) * | a) study controls for age * | b) study controls for any additional factor * | a) secure record (e.g. surgical records) * | a) yes * | b) non-respondents described | 7 |
| Goldstein et al., 2000 | a) yes with independent validation * | a) consecutive or obviously representative series of cases * | a) community controls * | a) no history of disease (endpoint) * | a) study controls for age * | No discussion of potential confounders/controls | a) secure record (e.g. surgical records) * | a) yes * | unable to calculate | 7 |
| Spitzer et al., 1998 | b) yes e.g. record linkage or based on self reports | b) potential for selection biases or not stated | b) hospital controls | a) no history of disease (endpoint) * | potential confounders/controls | b) study controls for any additional factor * | b) structured interview where blind to case/control status * | a) yes * | unable to calculate | 4 |
| Wood et al., 1998 | a) yes with independent validation * | b) potential for selection biases or not stated | b) hospital controls | a) no history of disease (endpoint) * | a) study controls for age * | b) study controls for any additional factor * | a) secure record (e.g. surgical records) * | a) yes * | a) same rate for both groups * | 7 |
| Steffen-Klatt et al., 2019 | a) yes with independent validation * | b) potential for selection biases or not stated | a) community controls * | a) no history of disease (endpoint) * | Did not control age | No discussion of potential confounders/controls | a) secure record (e.g. surgical records) * | a) yes * | unable to calculate | 5 |
| Martino et al., 2017 | a) yes with independent validation * | b) potential for selection biases or not stated | b) hospital controls | a) no history of disease (endpoint) * | a) study controls for age * | b) study controls for any additional factor * | a) secure record (e.g. surgical records) * | a) yes * | unable to calculate | 6 |
| Kienle et al., 2017 | a) yes with independent validation * | a) consecutive or obviously representative series of cases * | a) community controls * | a) no history of disease (endpoint) * | No discussion of potential confounders/controls | No discussion of potential confounders/controls | a) secure record (e.g. surgical records) * | a) yes * | unable to calculate | 6 |
| Stins et al., 2014 | a) yes with independent validation * | b) potential for selection biases or not stated | c) no description | b) no description of source | a) study controls for age * | b) study controls for any additional factor * | a) secure record (e.g. surgical records) * | a) yes * | unable to calculate | 5 |
| Moene et al., 2001 | a) yes with independent validation * | b) potential for selection biases or not stated | a) community controls * | a) no history of disease (endpoint) * | Did not control age | No discussion of potential confounders/controls | b) structured interview where blind to case/control status * | a) yes * | b) non-respondents described | 5 |
| Litwin & Cardeña, 2001 | a) yes with independent validation * | b) potential for selection biases or not stated | b) hospital controls | b) no description of source | No discussion of potential confounders/controls | No discussion of potential confounders/controls | a) secure record (e.g. surgical records) * | a) yes * | unable to calculate | 3 |
| Scévola et al., 2013 | a) yes with independent validation * | a) consecutive or obviously representative series of cases * | b) hospital controls | a) no history of disease (endpoint) * | No discussion of potential confounders/controls | No discussion of potential confounders/controls | a) secure record (e.g. surgical records) * | a) yes * | unable to calculate | 5 |
| Alper et al., 1997 | a) yes with independent validation * | a) consecutive or obviously representative series of cases * | b) hospital controls | a) no history of disease (endpoint) * | a) study controls for age * | b) study controls for any additional factor * | a) secure record (e.g. surgical records) * | a) yes * | unable to calculate | 7 |
| Koreki et al., 2020 | a) yes with independent validation * | a) consecutive or obviously representative series of cases * | a) community controls * | a) no history of disease (endpoint) * | a) study controls for age * | b) study controls for any additional factor * | a) secure record (e.g. surgical records) * | a) yes * | unable to calculate | 8 |
| Gerhardt et al., 2021 | a) yes with independent validation * | a) consecutive or obviously representative series of cases * | a) community controls * | a) no history of disease (endpoint) * | Did not control age | No discussion of potential confounders/controls | b) structured interview where blind to case/control status * | a) yes * | unable to calculate | 6 |
| Herrero et al., 2020 | a) yes with independent validation * | b) potential for selection biases or not stated | a) community controls * | a) no history of disease (endpoint) * | a) study controls for age * | b) study controls for any additional factor * | b) structured interview where blind to case/control status * | a) yes * | unable to calculate | 7 |
| Irorutola et al., 2020 | a) yes with independent validation * | a) consecutive or obviously representative series of cases * | a) community controls * | a) no history of disease (endpoint) * | Did not control age | b) study controls for any additional factor * | b) structured interview where blind to case/control status * | a) yes * | unable to calculate | 7 |
| Jungilligens et al., 2020 | a) yes with independent validation * | b) potential for selection biases or not stated | a) community controls * | a) no history of disease (endpoint) * | a) study controls for age * | b) study controls for any additional factor * | b) structured interview where blind to case/control status * | a) yes * | unable to calculate | 7 |
| Mousa et al., 2021 | a) yes with independent validation * | a) consecutive or obviously representative series of cases * | a) community controls * | a) no history of disease (endpoint) * | a) study controls for age * | b) study controls for any additional factor * | b) structured interview where blind to case/control status * | a) yes * | b) non-respondents described | 8 |
| Nisticò et al., 2020 | a) yes with independent validation * | a) consecutive or obviously representative series of cases * | a) community controls * | a) no history of disease (endpoint) * | No discussion of potential confounders/controls | No discussion of potential confounders/controls | b) structured interview where blind to case/control status * | a) yes * | unable to calculate | 6 |
| Ozdemir et al., 2020 | b) yes e.g. record linkage or based on self-reports | a) consecutive or obviously representative series of cases * | a) community controls * | a) no history of disease (endpoint) * | Did not control age | No discussion of potential confounders/controls | b) structured interview where blind to case/control status * | a) yes * | unable to calculate | 5 |
| Pick et al., 2020 | a) yes with independent validation * | a) consecutive or obviously representative series of cases * | a) community controls * | a) no history of disease (endpoint) * | Did not control age | No discussion of potential confounders/controls | b) structured interview where blind to case/control status * | a) yes * | unable to calculate | 6 |
| Wells, G.A., et al., *The Newcastle-Ottawa Scale (NOS) for assessing the quality of nonrandomised studies in meta-analyses*. 2000, Oxford. | | | | | | | | | | |

### Supplementary Table 5: Adapted Newcastle Ottawa Scale Ratings for Cross-sectional Studies

| Adapted Newcastle Ottawa Scale Ratings for Cross-sectional Studies | | | | | | | | | |
| --- | --- | --- | --- | --- | --- | --- | --- | --- | --- |
|  | Selection | | | | Comparability | | Outcome | | Total Score (0-8) |
|  | Representativeness of the sample | Sample Size | Ascertainment of exposure | Non-respondents | Comparability (age) | Comparability (other factors) | Assessment of outcome | Statistical test |  |
| Williams et al., 2019 | b) somewhat representative of the average in the target population (non-random sampling) * | b) not justified | a) validated measurement tool* | b) the response rate is unsatisfactory or the comparability is unsatisfactory no description of response rate | does not control for age | study does not control for additional factors | c) self-report* | a) The statistical test used to analyse the data is clearly described and appropriate and the measurement of the association is presented including confidence intervals or probability level (p-value) * | 3 |
| Myers et al., 2017 | a) Truly representative of the average in the target population (all subjects or random sampling)* | b) not justified | a) validated measurement tool* | b) the response rate is unsatisfactory or the comparability is unsatisfactory no description of response rate | does not control for age | study does not control for additional factors | c) self-report* | a) The statistical test used to analyse the data is clearly described and appropriate and the measurement of the association is presented including confidence intervals or probability level (p-value)* | 4 |
| Walther et al., 2019 | a) Truly representative of the average in the target population (all subjects or random sampling)* | b) not justified | a) validated measurement tool* | b) the response rate is unsatisfactory or the comparability is unsatisfactory no description of response rate | does not control for age | study does not control for additional factors | c) self-report* | a) The statistical test used to analyse the data is clearly described and appropriate and the measurement of the association is presented including confidence intervals or probability level (p-value)* | 3 |
| Boesten, Myers & Wijnen, 2018 | a) Truly representative of the average in the target population (all subjects or random sampling)* | b) not justified | a) validated measurement tool* | a) Comparability between respondents and non-respondents characteristics is established | does not control for age | study does not control for additional factors | c) self-report* | a) The statistical test used to analyse the data is clearly described and appropriate and the measurement of the association is presented including confidence intervals or probability level (p-value)* | 4 |
| Akyüz et al., 2017 | b) somewhat representative of the average in the target population (non-random sampling)* | b) not justified | b) non-validated measurement tool but the tool is available or described* | b) the response rate is unsatisfactory | does not control for age | study does not control for additional factors | c) self-report* | a) The statistical test used to analyse the data is clearly described and appropriate and the measurement of the association is presented including confidence intervals or probability level (p-value)* | 4 |
| Yayla et al., 2015 | b) somewhat representative of the average in the target population (non-random sampling)* | b) not justified | b) non-validated measurement tool but the tool is available or described* | b) the response rate is unsatisfactory or the comparability is unsatisfactory no description of response rate | does not control for age | study does not control for additional factors | c) self-report* | a) The statistical test used to analyse the data is clearly described and appropriate and the measurement of the association is presented including confidence intervals or probability level (p-value)* | 3 |
| Cohen et al., 2014 | a) Truly representative of the average in the target population (all subjects or random sampling)* | a) justified and satisfactory (power calculation)* | a) validated measurement tool* | b) the response rate is unsatisfactory or the comparability is unsatisfactory no description of response rate | does not control for age | study does not control for additional factors | c) self-report* | a) The statistical test used to analyse the data is clearly described and appropriate and the measurement of the association is presented including confidence intervals or probability level (p-value)* | 5 |
| Evren & Suat, 2007 | c) selected group of users | b) not justified | b) non-validated measurement tool but the tool is available or described* | b) the response rate is unsatisfactory or the comparability is unsatisfactory no description of response rate | does not control for age | study does not control for additional factors | c) self-report* | a) The statistical test used to analyse the data is clearly described and appropriate and the measurement of the association is presented including confidence intervals or probability level (p-value)* | 3 |
| Baillés et al., 2004 | a) Truly representative of the average in the target population (all subjects or random sampling)* | b) not justified | a) validated measurement tool* | b) the response rate is unsatisfactory or the comparability is unsatisfactory no description of response rate | does not control for age | study does not control for additional factors | c) self-report* | a) The statistical test used to analyse the data is clearly described and appropriate and the measurement of the association is presented including confidence intervals or probability level (p-value)* | 4 |
| Guz et al., 2003 | a) Truly representative of the average in the target population (all subjects or random sampling)* | b) not justified | a) validated measurement tool* | b) the response rate is unsatisfactory or the comparability is unsatisfactory no description of response rate | does not control for age | study does not control for additional factors | c) self-report* | a) The statistical test used to analyse the data is clearly described and appropriate and the measurement of the association is presented including confidence intervals or probability level (p-value)* | 4 |
| Tezcan et al., 2003 | c) selected group of users | b) not justified | b) non-validated measurement tool but the tool is available or described* | a) Comparability between respondents and non-respondents characteristics is established and the response rate is satisfactory* | does not control for age | study does not control for additional factors | c) self-report* | b) statistical test inappropriate/incomplete | 3 |
| Mitchell, Ali & Cavanna, 2012 | a) Truly representative of the average in the target population (all subjects or random sampling)* | b) not justified | a) validated measurement tool* | b) the response rate is unsatisfactory or the comparability is unsatisfactory no description of response rate | does not control for age | study does not control for additional factors | c) self-report* | a) The statistical test used to analyse the data is clearly described and appropriate and the measurement of the association is presented including confidence intervals or probability level (p-value)* | 4 |
| Holper et al 2021 | b) somewhat representative of the average in the target population (non-random sampling)* | a) justified and satisfactory (power calculation)* | a) validated measurement tool* | a) Comparability between respondents and non-respondents characteristics is established and the response rate is satisfactory* | does not control for age | study does not control for additional factors | d) no description | a) The statistical test used to analyse the data is clearly described and appropriate and the measurement of the association is presented including confidence intervals or probability level (p-value)* | 5 |
| Martino et al. 2020 | b) somewhat representative of the average in the target population (non-random sampling)* | b) not justified | a) validated measurement tool* | a) Comparability between respondents and non-respondents characteristics is established and the response rate is satisfactory* | does not control for age | study does not control for additional factors | c) self-report* | a) The statistical test used to analyse the data is clearly described and appropriate and the measurement of the association is presented including confidence intervals or probability level (p-value)* | 5 |
| Sarudiansky et al 2020 | b) somewhat representative of the average in the target population (non-random sampling)* | b) not justified | a) validated measurement tool* | b) the response rate is unsatisfactory or the comparability is unsatisfactory no description of response rate | does not control for age | study does not control for additional factors | c) self-report* | a) The statistical test used to analyse the data is clearly described and appropriate and the measurement of the association is presented including confidence intervals or probability level (p-value)* | 4 |
| Moskalewicz, A. and M. Oremus, *No clear choice between Newcastle–Ottawa Scale and Appraisal Tool for Cross-Sectional Studies to assess methodological quality in cross-sectional studies of health-related quality of life and breast cancer.* Journal of clinical epidemiology, 2020. **120**: p. 94-103.  Wells, G.A., et al., The Newcastle-Ottawa Scale (NOS) for assessing the quality of nonrandomised studies in meta-analyses. 2000, Oxford. | | | | | | | | | |

### Supplementary Table 6: Newcastle Ottawa Scale Ratings for Cohort Studies

| Newcastle Ottawa Scale Ratings for Cohort Studies | | | | | | | | | | |
| --- | --- | --- | --- | --- | --- | --- | --- | --- | --- | --- |
|  | Selection | | | | Comparability | | Outcome | | | Total Score (0-9) |
|  | Representativeness of the exposed cohort | Selection of the non exposed cohort | Ascertainment of exposure | Demonstration that outcome of interest was not present at start of study | Comparability of cohorts on the basis of the design or analysis (age) | Comparability of cohorts on the basis of the design or analysis (other factors) | Assessment of outcome | Was follow-up long enough for outcomes to occur | Adequacy of follow up of cohorts |  |
| Bodde et al., 2006 | b) somewhat representative of the average FND patient in the community * | c) no description of the derivation of the non-exposed cohort | a) secure record (e.g. surgical records) * | b) no | no mention of controls/confounds | no mention of controls/confounds | c) self-report | a) yes (only one timepoint necessary) * | b) subjects lost to follow-up unlikely to introduce bias - small number lost < 5%(?) or good description * | 4 |
| Kuyk et al., 2007 | b) somewhat representative of the average FND patient in the community * | c) no description of the derivation of the non-exposed cohort | a) secure record (e.g. surgical records) * | a) yes* | no mention of controls/confounds | no mention of controls/confounds | c) self-report | a) yes (only one timepoint necessary) * | c) follow-up rate >5%(?) and no description of those lost * | 4 |
| Kienle et al., 2018 | a) truly representative of the average FND patient in the community * | a) drawn from the same community as the exposed cohort * | b) structured interview * | a) yes* | no mention of controls/confounds | no mention of controls/confounds | c) self-report | a) yes (only one timepoint necessary) * | b) subjects lost to follow-up unlikely to introduce bias - small number lost < 5%(?) or good description * | 6 |
| Cope et al., 2017 | a) truly representative of the average FND patient in the community * | c) no description of the derivation of the non-exposed cohort | b) structured interview * | a) yes* | no mention of controls/confounds | no mention of controls/confounds | c) self-report | a) yes (only one timepoint necessary) * | b) subjects lost to follow-up unlikely to introduce bias - small number lost < 5%(?) or good description * | 5 |
| Jalilianhasanpour et al., 2019 | a) truly representative of the average FND patient in the community * | c) no description of the derivation of the non-exposed cohort, | b) structured interview * | b) no | no mention of controls/confounds | no mention of controls/confounds | c) self-report | a) yes (only one timepoint necessary) * | c) follow-up rate >5%(?) and no description of those lost | 3 |
| Gagny et al., 2021 | a) truly representative of the average FND patient in the community * | c) no description of the derivation of the non-exposed cohort | a) secure record (e.g. surgical records) * | a) yes* | no mention of controls/confounds | no mention of controls/confounds | c) self-report | a) yes (only one timepoint necessary) * | c) follow-up rate >5%(?) and no description of those lost | 4 |
| Wells, G.A., et al., *The Newcastle-Ottawa Scale (NOS) for assessing the quality of nonrandomised studies in meta-analyses*. 2000, Oxford. | | | | | | | | | | |

### Supplementary Figure 1: Funnel plot of SDQ-20 scores

**
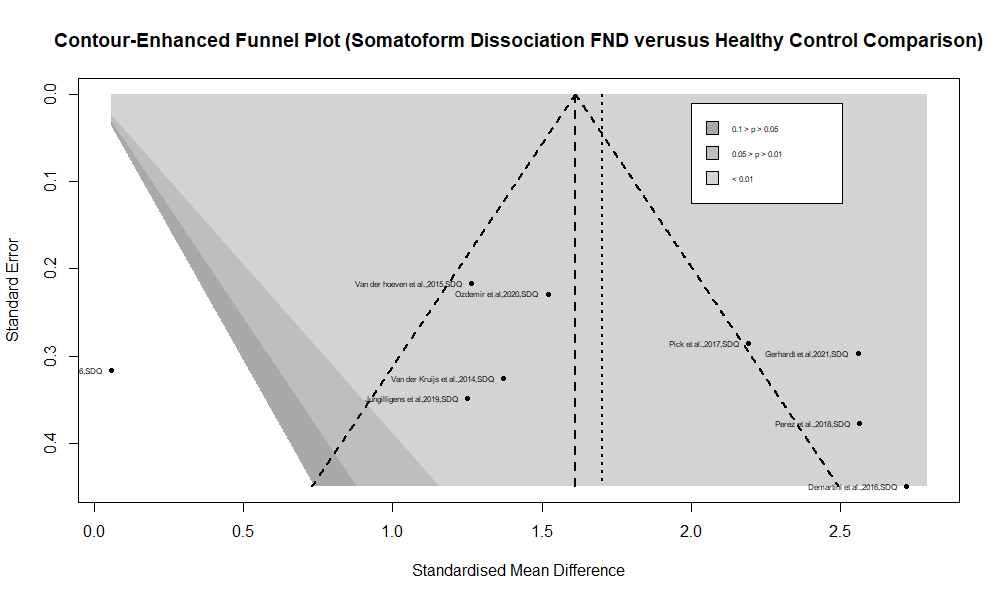
**

### Supplementary Figure 2: Forest plot of SDQ-20 scores with Demartini et al. removed


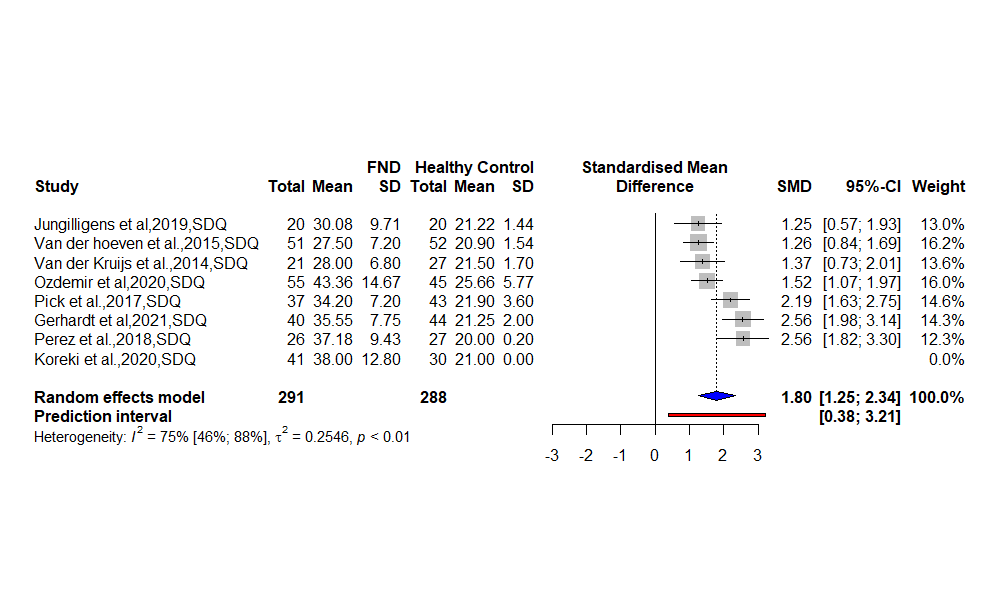


### Supplementary Figure 3: Funnel plot of SDQ-20 scores with Demartini et al. removed


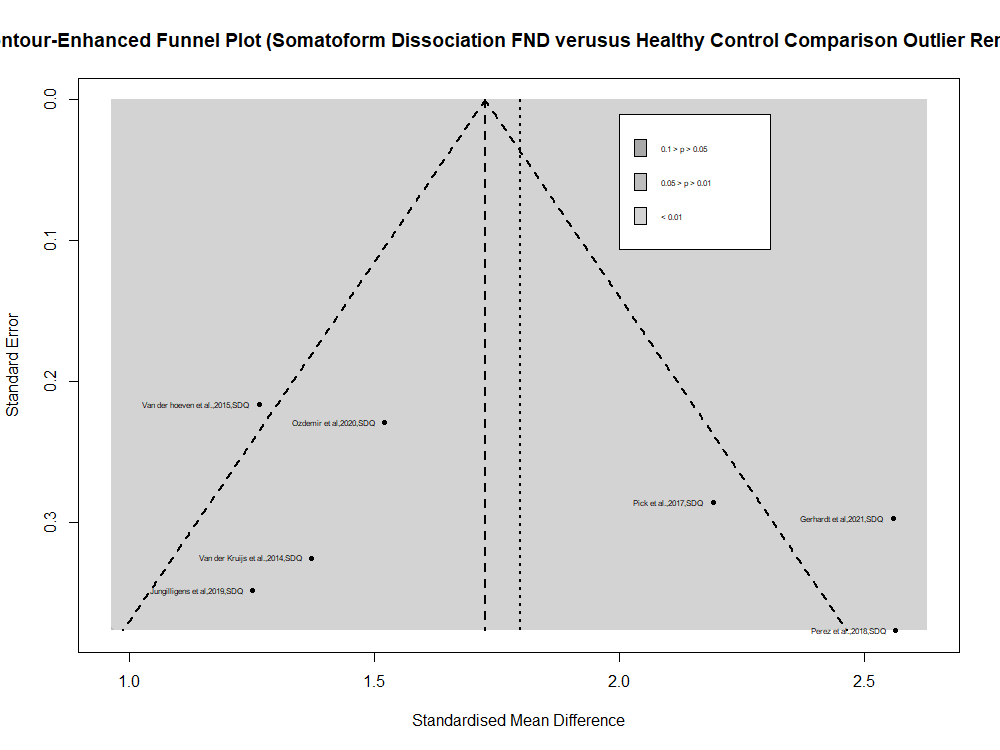


### Supplementary Figure 4: Funnel plot of Psychoform Dissociation Studies


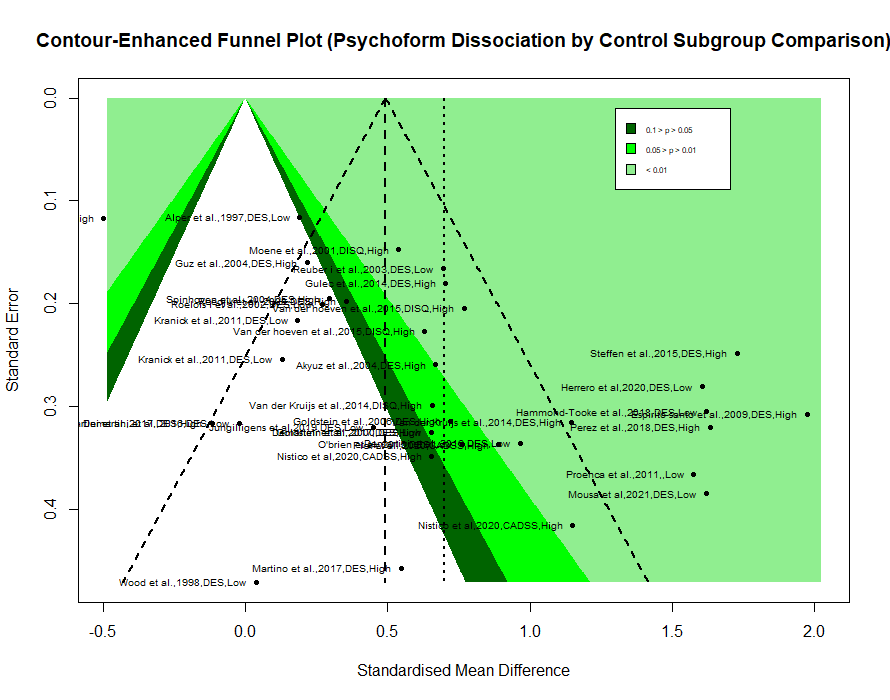


### Supplementary Figure 5: Funnel plot for Psychoform Dissociation in FND-seizure Vs FND-motor subgroups


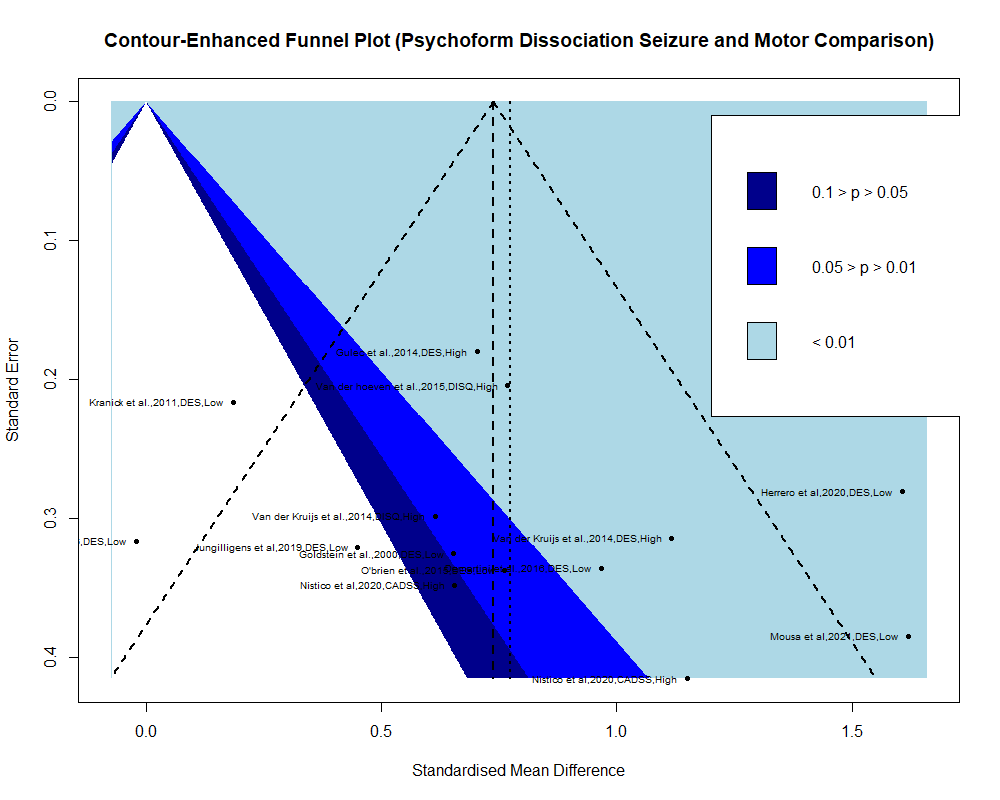

Supplement: Supplementary file 1 [file S205647242200597Xsup001.docx]
